# Internet gaming disorder scale: A comparison of symptoms prevalence, structure, and invariance in 12 nationally representative European adolescent samples

**DOI:** 10.1556/2006.2025.00090

**Published:** 2025-11-05

**Authors:** Andrea Stašek, Tommaso Galeotti, Natale Canale, Regina van den Eijnden, Daniela Husarová, Lukas Blinka

**Affiliations:** 1 Psychology Research Institute, Faculty of Social Studies, Masaryk University, Czech Republic; 2 Department of Developmental and Social Psychology, University of Padova, Italy; 3 Interdisciplinary Social Science, Utrecht University, The Netherlands; 4 Medical Education Centre, Faculty of Medicine, University of Pavol Jozef Šafárik Košice, Slovakia

**Keywords:** Internet Gaming Disorder, prevalence, psychometrics, network analysis, measurement invariance, HBSC

## Abstract

**Background:**

Internet Gaming Disorder (IGD) is recognized as a significant health issue in adolescents. However, the cross-national comparison and validation remain underrepresented in the literature. Thus, this study aimed to evaluate the symptom prevalence, dimensionality, and measurement invariance of the nine-item Internet Gaming Disorder Scale (IGDS) with data from the 2021–22 Health Behaviour in School-aged Children survey.

**Methods:**

Representative samples of adolescents aged 11–15 from 12 European regions (*N* = 44,126) were used. The IGDS was examined using network analyses and factor models with dynamic cut-offs.

**Results:**

Gaming intensity was more related to IGDS score than gaming frequency. Non-gaming boys at the time of measurement reported similar IGDS scores as daily gamers. All symptoms were more common in boys; Escapism and Preoccupation were the most common symptoms overall. A unidimensional structure for the IGDS across both genders and all regions was indicated. Only configural invariance was observed across genders, with notable higher roles for “Problems” and “Preoccupation” in boys, suggesting problematic direct gender comparisons. Measurement invariance suggested three relatively homogenous region groups, showing varying levels of invariance, and some groups achieving scalar invariance. Consequently, cross-regional comparisons should be approached with caution.

**Conclusions:**

The findings suggest large differences between boys and girls, moderate differences between age groups, and relatively high differences among regions.

## Introduction

Gaming engages around 32% of the world's population (Statista, 2024), and adolescents and young adults make up more than half of gamers. Although gaming can be beneficial (e.g., Halbrook, O’Donnell, & Msetfi, 2019; Snodgrass, Dengah, Polzer, & Else, 2019), a minority of gamers may suffer from an addiction-like disorder due to excessive gaming, which is acknowledged as Gaming Disorder (GD) by the 11th revision of the International Classification of Diseases (ICD-11 – WHO, 2019) and as Internet Gaming Disorder (IGD) in the appendix of the 5th revision of the Diagnostic and Statistical Manual of Mental Disorders (DSM-5 – APA, 2013). Thus, research has developed along two lines: early work relied on the DSM-5's polythetic IGD framework, while more recent studies adopt the ICD-11's monothetic GD criteria. The criteria are largely consistent (e.g., Yen, Chou, Liao, & Ko, 2023), though the DSM-5 captures a broader range of relevant symptoms. In this study, we primarily focus on IGD and IGD-related measures, while noting that IGD and GD overlap substantially but not fully.

Adolescents at risk of IGD and GD commonly exhibit symptoms related to using gaming as a means for escaping negative emotions, persistent preoccupation with gaming, increased tolerance for extended gaming sessions, and continued engagement despite experiencing negative consequences (King & Delfabbro, 2016; Király, Koncz, Griffiths, & Demetrovics, 2023). Furthermore, withdrawal symptoms can manifest when gaming is discontinued, leading individuals to prioritize gaming over other activities (Rehbein, Kliem, Baier, Mößle, & Petry, 2015; Xie & Tang, 2024). Researchers distinguish between “core” and “peripheral” symptoms (e.g., Charlton & Danforth, 2007) and, while the aforementioned symptoms usually belong to the “peripheral” dimension, the negative consequences of gaming (i.e., conflict with parents, problems with school, hiding one's gaming behavior; Colder Carras & Kardefelt-Winther, 2018; Peeters, Koning, Lemmens, & Eijnden, 2019) appear to be the “core” criteria for differentiating between recreational and problematic gamers. However, the suggested symptoms have their own specifics that further complicate simple categorization.

Escapism is a particularly illustrative case because of its dual and context-dependent role in gaming and gaming disorder. It is related to coping mechanisms, mood and affect regulation, needs and motives — why any medium, including video games, is used in the first place (Stevens & Dillman Carpentier, 2017). At the same time, it has been linked to both adaptive and maladaptive outcomes (e.g., Hussain, Jabarkhail, Cunningham, & Madsen, 2021; Stenseng, Falch-Madsen, & Hygen, 2021) and shown to both precede and reinforce problematic gaming (e.g., Krossbakken et al., 2018; Melodia, Canale, & Griffiths, 2022), because it is theoretically embedded within frameworks, such as the Interaction of Person-Affect-Cognition-Execution (I-PACE) model (Brand et al., 2019), the gratification-compensation approach (Wegmann et al., 2025), and the Compensatory-Dissociative Online Gaming model (Giardina et al., 2024). This dual and context-dependent role makes escapism a clear example of how IGD symptoms evade straightforward classification.

Research clearly suggests a gender disparity in IGD symptoms, with boys reporting higher symptomatology than girls (Andreeta et al., 2020; Király et al., 2023; Van der Neut, et al., 2023). Moreover, countries within Europe and globally differ in the prevalence of IGD (Müller et al., 2015; Thomas, Gaspar, Al Beyahi, Al Bassam, & Aljedawi, 2024). The reported prevalence of IGD varies across studies, largely due to the differing methodologies and instruments employed in measuring the disorder (Stevens, Dorstyn, Delfabbro, & King, 2021; Van der Neut et al., 2023).

One of the most widely adopted scales for assessing IGD is the nine-item Internet Gaming Disorder Scale (IGDS; Lemmens, Valkenburg, & Gentile, 2015), which is based on the nine criteria of DSM-5. The IGDS developers and other research teams (e.g., Wartberg, Kriston, & Thomasius, 2017, 2020) found gender and age differences in the IGDS scores, with men/boys scoring higher than women/girls and adolescents scoring higher than older age groups. Overall, the IGDS has been considered one of the better-functioning measurements, with satisfactory reliability and validity when compared to other measures (Anthony, Mills, & Nower, 2023; Karhulahti, Martončik, & Adamkovič, 2023; King et al., 2020) and when systematically tested in an international meta-analysis (Gisbert-Pérez, Martí-Vilar, Merino-Soto, & Vallejos-Flores, 2022). However, there are also several limitations. First, although the IGDS has been validated in many countries, e.g., Germany (Wartberg, Kriston, & Thomasius, 2020), Romania (Coşa, Dobrean, & Balazsi, 2024), and Turkey (Evren, Dalbudak, Topcu, Kutlu, & Evren, 2017), and was used in international projects like HBSC (Van der Neut et al., 2023; Boniel-Nissim et al., 2024), no cross-national validation has been conducted to date. Second, previous validation studies primarily relied on statistical models such as factor analysis, which has several drawbacks (discussed below). To our knowledge, there is no research that uses network psychometrics (Christensen & Golino, 2021; Golino et al., 2020; Jamison, Golino, & Christensen, 2022; Jones, Mair, Simon, & Zeileis, 2020; Van Borkulo et al., 2023) to examine the symptom structure of the IGD on the international level. While in the latent model, a specific cause (i.e., IGD) gives rise to otherwise independent symptoms, the network approach posits that symptoms mutually and causally affect each other, forming a complex system. Evidence suggests that problematic gaming symptoms are indeed mutually dependent (e.g., Karhulahti, Behm, & Lukka, 2023; Karhulahti, Siutila, Vahlo, & Koskimaa, 2023; Nalwoga, Kizito, Kigongo, Atwine, & Kabunga, 2024; Petrovskaya, 2022; Sun, Cash, Mullen, & Rae, 2023); therefore, we believe that IGD symptomatology aligns with the network approach.

The current study aimed to assess the prevalence, dimensionality, and invariance of the IGDS, primarily across regions and genders. Using representative data from 12 regions of Europe (i.e., Cyprus, Czechia, England, Estonia, Iceland, Malta, North Macedonia, the Netherlands, Scotland, Serbia, Slovakia, and Slovenia), we employed the network approach to symptomatology (e.g., Borsboom & Cramer, 2013; Robinaugh, Hoekstra, Toner, & Borsboom, 2020) to analyze the IGDS at the symptom level. Based on the identified issues of the current literature, this study seeks to answer the following questions:

1. What is the prevalence of IGD symptoms (per region, gender, and age group)?
2. What dimensions does the IGDS have?
3. Is the IGDS invariant across countries and genders?

The existing findings suggest that boys generally play video games more than girls. Moreover, gaming does not change with age in boys and it decreases as girls age. Based on existing findings (Gomez, Stavropoulos, Tullett-Prado, Schivinski, & Chen, 2022; Lecuona, Lin, Montag, Pontes, & Pakpour, 2024; Liu et al., 2022; Stašek, Blinka, & Stavropoulos, 2024), we hypothesized two dimensions/clusters (H1); however, to our knowledge, no study has tested the dimensionality in a nationally representative adolescent population with network approaches. We hypothesized that the dimensions (H2) were cognitive-emotional (i.e., Preoccupation, Tolerance, Withdrawal, and Escape) and behavioral-consequential (i.e., Persistence, Problems, Deception, Displacement, and Conflict), and that (H3) the Escape symptom was the bridge. Theoretically, we do not expect gaming disorder symptoms to function differently across the examined regions (H4) because popular games tend to be similar worldwide due to the globalization of the gaming market, despite some empirical findings from different kinds of data (De Palo et al., 2019; Király et al., 2019; Poon et al., 2021; Stavropoulos et al., 2018). On the other hand, we hypothesized IGDS to be non-invariant across genders (H5): boys/men are much more likely to engage in gaming than girls/women (Fam, 2018; Kim et al., 2022; Stevens et al., 2021) and gender-based differences in gaming motivations (Laconi, Pirès, & Chabrol, 2017) and gaming behavior (Davies, Headleand, & Hicks, 2020) have been consistently reported.

## Methods

### Participants

The initial sample included 12 regions (*n* = 68,336). Adolescents who did not respond to at least three of the gaming symptom questions were excluded from the sample. We decided on the three missing response criteria to ensure the validity of the answers. Table S1 in the Supplementary Material [SM] shows additional information on each region's missing data. The final sample of 44,126 respondents was gender-balanced (54.7% boys, 44.8% girls, 0.5% missing) and age-balanced (31.6% 11 years old, 36.7% 13 years old, 31.7% 15 years old).

### Measures

#### Internet gaming disorder scale

The symptoms of Internet Gaming Disorder (IGD) were measured with the dichotomous (i.e., yes/no) nine-item Internet Gaming Disorder Scale (Lemmens et al., 2015). With these nine items, participants indicated whether they experienced the following symptoms during the preceding year: Preoccupation, Tolerance, Withdrawal, Persistence, Escape, Problems, Deception, Displacement, and Conflict. The wording of each symptom is in the SM (Table S2).

#### Gaming frequency and intensity

A single item measured the frequency of gaming (“How often do you play games?”) and the options were “(Almost) never”, “Less than one day a week”, “1 day a week”, “2 or 3 days a week”, “4 or 5 days a week”, and “(Almost) every day”. The intensity (i.e., time per gaming session) was measured by the item “On a day that you play games, how much time do you spend gaming?” with the options “1–2 h”, “2–4 h”, “4–6 h”, “6–8 h”, and “8 h or more”.

### Sampling procedure

The study presents data collected in 2021–22 from the international Health Behaviour in School-aged Children (HBSC) study, which is conducted every four years in 50 countries in Europe and North America in collaboration with the WHO Regional Office for Europe. The HBSC aims to monitor health and health-related behavior among 11-, 13-, and 15-year-old adolescents in their social context. The present study included 12 countries that applied the optional package with the IGDS measure. National representative samples of adolescents aged 11 to 15 from randomly selected schools and classes in each country were recruited as the primary sampling units. Next, passive or active parental-informed consent was obtained, depending on national ethics regulations. The data were collected by self-reported standardized online or paper questionnaires administered in school settings. Participation in the data collection was voluntary, and respondents were informed about their answers' confidentiality. All questions were translated from English into the national languages by national research teams and then back-translated into English to ensure semantic equivalence. The study's methodology is described in the HBSC protocol and it is strictly followed by all of the countries (Inchley, Currie, Samdal, Jåstad, Cosma, & Nic Gabhainn, 2023). More detailed information about the study is available at www.hbsc.org.

### Analytical procedure

The data were analyzed with R/RStudio software (version 4.3.2; R Development Core Team, 2011). The preregistered psychometric procedure and analytical script are publicly available at osf.io.

Upon receiving the official data from the HBSC, we selected data from the 12 regions that employed the gaming-related variables (*n* = 68,336). We estimated missing values for the variables of interest, ranging from Hours per session (43.92% cases missing) to Days per week (25.35% cases missing). Gender was missing in 1.04% of the cases. The variable Days per week was used as a filter question in Czechia, England, Estonia, Scotland, Slovakia, and Slovenia. In these countries, adolescents who responded to “(Almost) never” play games did not answer to the IGDS. The IGDS items were missing in 34.97–35.37% of the cases. Then, cases with three or more missing IGDS items were excluded to ensure valid responses, resulting in 0.21–0.46% of responses that were missing for each IGDS item and *n* = 44,126 (gender missing in 0.47% cases). This sample was used in all analyses that described or tested the full sample (i.e., pooled data) or regional groups to maximize representativity. Wherever gender was considered (e.g., comparing gender groups), cases with a missing gender response were excluded, resulting in *n* = 43,918. Missing responses and the progress of deletion are described in Table S2 in the SM. In general, all respondents with valid answers were used, including those who reported zero symptoms, which allowed us to gain evidence about the whole population, specifically about the mutual alignment of symptoms (e.g., the likelihood of not having Symptom A when Symptom B is not present).

The prevalence of symptoms was examined with basic descriptive statistics, chi-square tests, and Cramer V (large: *V* > 0.15; moderate: 0.10 ≤ *V* < 0.15; small: *V* < 0.10). Psychometric dimensionality was checked by the Exploratory Graph Analysis (EGA; Golino et al., 2020; Jamison et al., 2022) with the graphical least absolute shrinkage and selection operator (GLASSO) estimated for 1) the full sample, 2) each gender, 3) each region, and 4) each gender in each region. Within metric network invariance, we applied the method only to test gender invariance and applied the Benjamini-Hochberg procedure (Benjamini & Hochberg, 1995) for multiple comparisons and the 0.05 alpha level (Jamison et al., 2022). Next, we explored invariance across regions using Network Trees (Jones et al., 2020). This analysis assesses which grouping variable (i.e., age, gender, or region) contributes to the largest heterogeneity in the symptom network. The resulting network tree graph shows partitioning based on invariance and hierarchy – the variable that causes the largest heterogeneity to be at the top level/the first split. We have applied the 0.01 alpha level to this analysis and tested the nine-item IGDS network structures across regions (12 in total), genders (boys and girls), and ages (11, 13, and 15 years old).

Next, we freely estimated the networks for each gender and region (resulting in 24 network models) with the Ising estimator (Van Borkulo et al., 2014). In the Ising model, the *edges* (i.e., connections between the variables – *nodes*) are equivalent to logistic regression coefficients and indicate the so-called *alignment*. Alignment represents the likelihood of having the same value (i.e., two symptoms not present or two symptoms present; Van Borkulo et al., 2014). The edge weights are an average of logistic regression coefficients between two nodes (i.e., *variableA* → *variableB* and *variableB* → *variableA*). The stability of the edges is presented in the SM (*bootnet* package; Epskamp et al., 2018; Figures S4–S27).

Finally, using the maximum likelihood (ML) estimator, we calculated the factor models of the IGDS for 1) the full sample, 2) each gender, 3) each country, and 4) each gender in each country. This method was included for two reasons: (i) factor invariance is the go-to method for most researchers, and, since the network and factor models are mutually dependent, (ii) the factor approach allows us to check and confirm the obtained network results. We applied the novel dynamic fit index approach (McNeish & Wolf, 2023) to evaluate the model fit indices that exceed the traditionally used cut-offs (Hu & Bentler, 1999), which lack generalizability. When comparing constrained models within measurement invariance, the relative change in the model fits was assessed traditionally because dynamic cut-offs have not yet been developed. Rutkowski and Svetina (2014) suggested model fits in large samples and many groups as “good” when ΔCFI and ΔTLI ≤ 0.02 and ΔRMSEA ≤ 0.03.

### Ethics

This study was conducted in accordance with the Declaration of Helsinki. All participants were informed about the study, parental consent was obtained, and the data collection was approved by the local institutional review boards (Inchley et al., 2023).

## Results

### Prevalence of symptoms

**Fig. 1.**
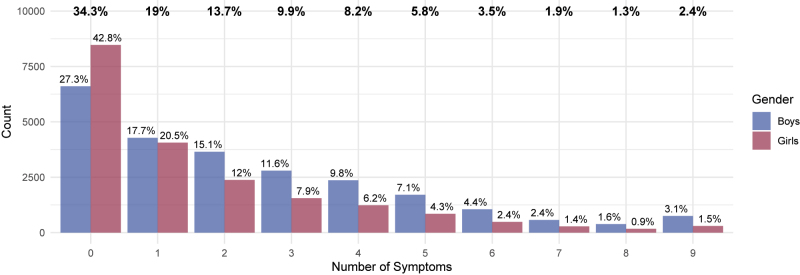
Distribution of symptom prevalence *Notes*. The percentages of boys (*n* = 24,149) are blue, and the percentages of girls (*n* = 19,769) are red. The row of percentage values on the top in bold denotes the occurrence of symptoms in the two groups together (*N* = 43,918). The gender samples in the figure excluded cases that did not respond to the gender item.

Figure 1 presents the distribution of the number of adolescents who reported experiencing zero to nine symptoms in the preceding year. A total of 34.3% participants reported no symptoms. There were evident gender differences in the prevalence; 63.3% of girls (in contrast to 45% of boys) reported zero or one symptom. Consequently, more often than girls, boys reported two or more symptoms. The SM offers detailed frequencies per symptoms for each gender, region, and age (Table S3), and the overall prevalence of the risk of developing IGD for each region and gender (Figure S1). The prevalence of risk based on the data used in this study has been previously published in the 2024 HBSC report (Boniel-Nissim et al., 2024), however, the SM offer a more detailed overview. In Table 1, the average number of symptoms per region and gender is reported.

**Table 1.** Average symptom occurrence per region and gender (ordered by boys' mean)

Region	Boys		Girls		Total	
	*M*	*n*	*M*	*n*	*M*	*n*
1. Malta (MT)	3.09	1,601	2.27	1,432	2.7	3,064
2. North Macedonia (MK)	3.03	1,934	2.03	2,113	2.58	3,141
3. England (ENG)	2.91	1,683	2.22	1,440	2.51	4,050
4. Scotland (SCT)	2.77	1,200	1.67	943	2.3	1,708
5. Slovakia (SK)	2.73	993	1.69	715	2.29	2,172
6. Estonia (EE)	2.56	2,166	1.81	1,524	2.26	3,720
7. Cyprus (CY)	2.46	2,162	1.52	2,252	1.98	4,435
8. Serbia (RS)	2.28	1,346	1.39	1,078	1.88	2,424
9. Iceland (IS)	2.17	1,038	1.00	785	1.8	1,896
10. Czechia (CZ)	2.12	5,324	1.39	4,006	1.7	9,330
11. Slovenia (SI)	1.99	2,613	1.26	2,039	1.67	4,655
12. Netherlands (NL)	1.74	2,089	0.99	1,442	1.43	3,531
Total	2.40	24,149	1.59	19,769	2.04	44,126

*Note.* The total column includes participants who had a missing value on gender.

**Fig. 2.**
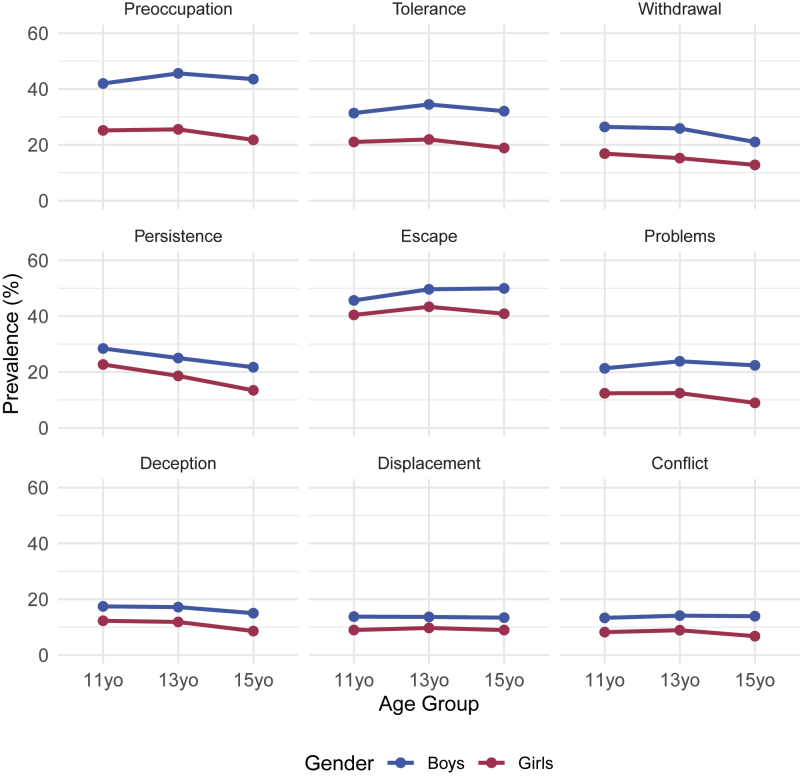
Prevalence of IGD symptoms by age group *Note*. The gender samples in the figure excluded cases that did not respond to the gender item.

In Fig. 2, we present the prevalence of the symptoms for each gender. We also tested the differences between 11-, 13-, and 15-year-olds in the prevalence of each symptom in each gender*region*age group (Table S3 in the SM). Across all groups, experiencing Escapism and Preoccupation in the last year was reported most frequently (48% of boys and 42% of girls; 44% of boys and 24% of girls), while Conflict was the least reported (14% of boys and 8% of girls). The largest gender differences were in the symptom of Preoccupation, while the largest age differences were in Persistence.

**Fig. 3.**
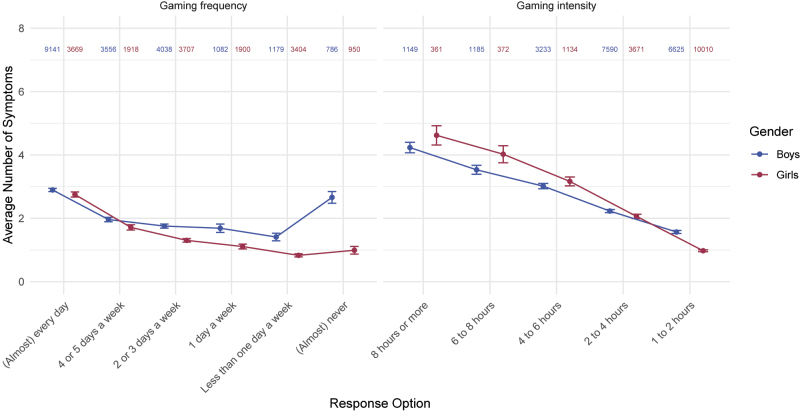
*Gaming frequency* (“How often do you play games?”) and *intensity* (“On a day that you play games, how much time do you spend gaming?”) in relation to the number of symptoms *Notes*. The color-coded numbers represent group sizes per response and gender. Points represent means, error bars represent their 95% confidence intervals. Only data from respondents who indicated their gender, gaming frequency, and gaming intensity were used in this graph.

The mean number of reported symptoms is lower in adolescents with a lower frequency of gaming (i.e., “How often do you play games?”) and intensity (“On a day that you play games, how much time do you spend gaming?”) (Fig. 3). Moreover, high-intensity gaming is connected to more symptoms on average than high-frequency gaming. No matter the frequency, adolescents gaming more than four hours per session usually report more symptoms (> 3) than daily gamers (< 3). Specifically, adolescents that dedicate eight and more hours to gaming per session, reported more than four symptoms on average. Daily gamers reported three symptoms on average.

Interestingly, we found a notable difference in the average number of symptoms between boys and girls who reported gaming “(Almost) never” per week. The mean symptom count was 2.66 (Md = 2; range = 0–9) in boys and 0.99 (Md = 0; range = 0–9) in girls. The gender differences are further reflected in the symptom patterns. In boys, the prevalence of individual symptoms is similar in both the *(almost) never* subgroup as compared to the whole boy sample (e.g., Escape 49.16% vs 48.86%; Preoccupation: 47.07% vs 44.97%; see Table S3). In girls, the prevalence of symptoms was notably lower in the *(almost) never* category compared to the whole girl sample (e.g., Escape 22.85% vs. 40.64%; Preoccupation 13.47% vs 24.24%).

### Dimensionality

The point-biserial correlations of the nine symptoms are in the SM (Table S4). The results of the EGA showed only one dimension in our data across all tested groups (full sample; two gender groups, 12 region, and 24 region*gender groups); thus, Hypothesis 1 about two dimensions was not supported. Furthermore, we re-examined the models with the CFA, which similarly supported the one-factor solution (Table 2 for full sample and gender groups; Table S5 in SM for region models); however, according to the dynamic fit cut-offs (available in SM, Table S6), the factor models did not usually have satisfactory fit (see level of misspecification).

**Table 2.** Goodness-of-fit indices for the full sample and gender models

Sample	*n*	*χ* ^2^	CFI	TLI	SRMR	RMSEA	RMSEA 90% CI		Level of misspecification^1^
							Lower	Upper	
Full sample	44,126	4810.478	0.946	0.928	0.035	0.064	0.063	0.066	Moderate
Gender									
Boys	24,149	2605.246	0.944	0.925	0.036	0.064	0.062	0.066	Moderate
Girls	19,769	2371.017	0.941	0.921	0.037	0.067	0.065	0.069	Moderate

*Notes*. All models had 18 parameters and 27 degrees of freedom. CFI = Comparative Fit Index. TLI = Tucker-Lewis Index. SRMR = Standardized root mean squared residual. RMSEA = Root mean squared error of approximation. CI = Confidence interval. Full sample included cases that did not indicate their gender. Gender samples excluded cases that did not respond to the gender item.

^1^This evaluation is based on McNeish and Wolf (2023). We estimated custom dynamic fit cut-offs of the CFI, SRMR, and RMSEA for each model. Level-1 cut-offs represent “small” misspecification, Level-2 cut-offs are a “moderate” misspecification, and Level-3 cut-offs are a “large” misspecification. When a model's fit indices reach different levels of misspecification, the average or most prevalent level is chosen (e.g., in the case of CFI - Level 1, SRMR - Level 2, and RMSEA - Level 2) and we evaluate the misspecification as moderate.

Due to the findings above, Hypotheses 2 and 3 were also not supported. We further explored the structures of the symptoms in each gender*region (two genders * 12 regions; Figure S2 in the SM). Although the global networks are unidimensional according to EGA, certain symptoms are often interconnected more closely than others. This varies significantly across the groups, and we cannot claim that IGDS splits into two or more clusters of symptoms in our adolescent data.

### Measurement invariance

On the *configural* level, the results showed that IGDS has a unidimensional (non-clustered) structure. The next step was the *metric* level of network invariance. Across gender, metric invariance was not achieved in the network tests (in line with Hypothesis 4) while reaching the *scalar* invariance in the factor-analytic approach (Table S7 in the SM). We lean towards the network approach due to our theoretical expectations for the symptoms' functioning and the misfit of factor models found earlier. Moreover, the network invariance test specified the non-invariant symptoms: the symptoms of Preoccupation and Problems were identified as *metrically *non-invariant across genders at the 0.05 alpha level. Therefore, we do not recommend simply comparing the IGDS scores across genders because it may lead to biased results.

To explore the metric invariance across regions and the structural differences in detail, we estimated a network tree to identify meaningful invariant groups in the data. The overall resulting partitioning is presented in SM (Figure S3). The splitting algorithm indicated that the most prominent source of heterogeneity (non-invariance) was regional: we labeled the groups as Regional Group 1 (RG1: Czechia, Iceland, the Netherlands, Serbia, and Slovenia) and Regional Group 2 (RG2: Cyprus, England, Estonia, Malta, North Macedonia, Slovakia, and Scotland). The second level of splits identified gender as the source of non-invariance in RG1 and RG2. The third level of splits was again regional and a) isolated the Netherlands in both boys and girls and b) divided RG2 in both genders into Cyprus, Estonia, and Slovakia (dubbed Regional Group 2a; RG2a) and North Macedonia, Malta, England, and Scotland (dubbed Regional Group 2b; RG2b). In the subsequent splits, the analysis also suggested age as the source of heterogeneity, especially in three other subgroups (i.e., Czech boys and girls, Icelandic boys), where 15-year-olds differed from the younger groups.

It is important to note that the network tree does not test invariance traditionally. The algorithm searches for the best data partitioning using the pre-selected covariates (i.e., region, gender, and age here) thus “*elucidating areas where heterogeneity is most impactful*” (Jones et al., 2020, p. 942).

The partitioning aligned with the symptom occurrence within the regions (Table 1 above). RG1 had a relatively lower occurrence of symptoms in both genders when compared to RG2. Within RG2, Malta, North Macedonia, England, and Scotland had the higher occurrence of symptoms in both genders in contrast to Slovakia, Estonia, and Cyprus.

Additionally, we tested the identified region groups for factor invariance and found mixed results across groups for invariance level and model fits: some groups reached only *configural *invariance, others *scalar *invariance (see Table S8 in the SM), and several models had a poor model fit. Researchers who wish to compare subgroups within these larger groups should be cautious; however, in the case of models that reach scalar invariance, the comparison should produce low or negligible bias.

## Discussion

This study explored symptom prevalence, dimensionality, and measurement invariance across genders and regions of the Internet Gaming Disorder Test (Lemmens et al., 2015) in 12 representative samples of European adolescents.

### Prevalence of symptoms

Preoccupation (i.e., only thinking about gaming) and Escape (i.e., playing to avoid thinking about annoying things) symptoms were the most frequent. These two criteria represent the cognitive-emotional aspects of IGD, as opposed to more behavioral-consequential symptoms, such as negative consequences, which our sample reported less frequently. This is in line with the “core” vs “peripheral” distinction of symptoms (Charlton & Danforth, 2007), emphasizing that core symptoms (i.e., negative consequences) are considered riskier, while others (i.e., Preoccupation and Escape) do not necessarily result in adverse outcomes (King et al., 2020). In detail, Preoccupation may reflect engagement driven by positive motivations rather than addiction, because more preoccupied adolescents could explore more game-related media and discuss it with friends (Petrovskaya, 2022). Thus, Preoccupation may signal a strong connection and mindful involvement with gaming, all activities associated with better well-being as suggested by the Dualistic Model of Passion (Vallerand, 2015). Similarly, prior research reports that Escape performs ineffectively in distinguishing IGD players from those without the disorder (Castro-Calvo et al., 2021; Király et al., 2017). Interestingly, the recently proposed Compensatory-Dissociative Online Gaming model (Giardina et al., 2024) suggests differentiating between active escapism, escape, and dissociation — the first being not inherently problematic (involving gamers leveraging video games to cope with life challenges), while the others represent a means to avoid undesirable physical environments. Thus, the aspect of escapism in general warrants further exploration in future studies. Our findings also highlight that Persistence (i.e., being unable to reduce game time) decreases with age. A recent review underlines that impaired control in gaming relates to deficits in self-control and alterations in the brain regions responsible for executive functions, reward processing, and inhibitory control (Kowalik & Delfabbro, 2025). Adolescent developmental changes include structural and functional changes in the brain that gradually enhance self-regulation abilities with age (Dahl, Allen, Wilbrecht, & Suleiman, 2018).

Gaming intensity and the frequency of IGD symptoms showed interesting relationships. Boys who “never or almost never” play games showed similar symptoms on average to those who play daily. These findings are surprising given that adolescents who do not play games are not expected to suffer negative consequences from them. The available data do not allow for an easy explanation of this result, but we can speculate that parental restrictions and withdrawal from being unable to play during data collection could play a role; nonetheless, we suggest that for girls, (almost) never gaming could really mean lower interest in gaming, while for boys, it represents less gaming than they wish (e.g., because they are restricted). The discrepancy in girls and boys also shows that the phenomenon could not be a result of response bias (e.g., misinterpretation, lack of attention checks): such an error would likely occur in both genders. Thus, further studies to incorporate low- or non-frequency gamers are needed. Finally, gaming intensity was positively associated with the reported number of symptoms. These findings align with previous research that reported that playing for long periods of time (i.e., more than four hours a day) is associated with the development of IGD (e.g., Jeong et al., 2021); however, the effect is likely indirect, more complex, and should not be treated as a criterion of IGD (Strojny et al., 2023). Nonetheless, long sessions were also previously linked to other health problems (Katz et al., 2024; Leung et al., 2024), potentially due to the displacement mechanism (Ballou, Hakman, Vuorre, Magnusson, & Przybylski, 2025). Therefore, in line with previous recommendations (Wegmann, Billieux, & Brand, 2022), we argue that gaming intensity should be considered in relation to the psychological factors that underlie maladaptive usage patterns, both in screening and in clinical research.

### Dimensionality

Despite our hypotheses, we found that the IGDS did not collapse into clusters (i.e., dimensions). Factor unidimensionality was established in some papers that use the IGDS and study adolescents (Coşa et al., 2024; Lemmens et al., 2015) and youth/adults (Evren et al., 2017; Lei et al., 2020); in other studies, the fit was not satisfactory (Paschke, Sack, & Thomasius, 2021). In our study, the fits of the tested factor models were unsatisfactory on a moderate or high level. It is important to note that even a relatively fitting factor model is not evidence for the general adequacy of the factor approach. It is the expected structure of the theoretical model (i.e., causal mechanism) that should be the incentive for choosing a methodology (Fried, 2020a, 2020b); thus, we stress that our factor-analytical results are only informative, and their problematic fit only shows the expected misspecification. Since IGD classifies nine *different* symptoms that mutually affect each other (e.g., Karhulahti, Behm, & Lukka, 2023; Karhulahti, Siutila, et al., 2023; Nalwoga et al., 2024; Petrovskaya, 2022; Sun et al., 2023), we lean towards the network results.

The found unidimensionality can be partly attributed to the age of our sample because adolescents are still developing coping mechanisms and emotional and behavioral regulation (Compas et al., 2017; Silvers, 2022). This results in the well-described high co-occurrence of problematic or risky adolescent behavior (Ellis et al., 2012; Jessor, 2017; Meader et al., 2016). Such a structure of mutual influence resembles a strongly interconnected network that is characterized by high activation, hysteresis, and vulnerability (Cramer et al., 2016; Van Bork, Lunansky, & Borsboom, 2024). Simply put, when one behavior (or symptom) occurs, it can quickly and easily reinforce other symptoms, which can then stay highly present (i.e., activated) for a relatively long time. Unlike in adults, adolescents' functioning can be described as a vulnerable state of more strongly interconnected symptoms due to their still-developing resilience, coping, and self-regulation.

### Measurement invariance

The configural level of invariance in IGDS was reached in all groups, similar to prior research (e.g., the Romanian version, Coşa et al., 2024). The metric invariance was not conclusively reached for gender due to the network invariance of the Preoccupation and Problems symptoms. Also, the boys' network, as opposed to the girls' one, resembled the hypothesized two-cluster structure of the cognitive-emotional (i.e., peripheral) and behavioral-consequential (i.e., core) symptoms – the boys' network had stronger edges within the two groups of symptoms than between the clusters.

The measurement across the regions was also not conclusively invariant. Therefore, the results for boys and girls drawn above from the pooled regional data should be interpreted cautiously. Even though metric invariance was reached in factor invariance tests, the model fits were unsatisfactory.

Upon closer examination and the suggestion of the network tree result (Figure S3), we obtained mixed results regarding invariance across three region groups – the Netherlands, Slovenia, Czechia, Iceland, and Serbia (RG1); Cyprus, Estonia, and Slovakia (RG2a); and Scotland, England, North Macedonia, and Malta (RG2b). Regional disparities in the prevalence of individual symptoms were found before (Fam, 2018; Gao, Wang, & Dong, 2022; Müller et al., 2015), with one explanation concerning the various regional cultures and norms for adolescents' leisure time. However, there is a lack of comparative studies done in the European context (Sauerwein & Rees, 2020), and the differences in organized leisure time activities were not conclusively attributed to region/country membership (Badura et al., 2021; Lareau, 2018; Meier, Hartmann, & Larson, 2018).

IGDS is a screening tool, so we can expect lower measurement precision than diagnostic tools (Borsboom, 2006): the data are highly heterogenic – unlike community samples, population data include a large proportion of participants with no symptoms whatsoever (34.2% in our case) and at the highest risk (2.4% of our respondents reported all symptoms). Therefore, there is much higher variability within groups (i.e., genders, regions) than between them. Furthermore, the number of parameters tested in international invariance studies is so high that achieving the equality of measurement at such a scale is almost unrealistic (Rutkowski & Svetina, 2014). We thus conclude that, given these high demands (i.e., invariance in international and culturally diverse populations), the IGDS functions relatively well.

### Limitations

Our study has several limits: we used data from 12 European regions that administered the IGDS. These regions are not representative of the entire continent of Europe. We also approached the regions and genders as relatively homogeneous groups, even though there are very likely further within-region and within-gender differences (e.g., based on the individual's socio-economic status or family relations). By pooling the data this way, we drew a rather big picture; however, we risk information reduction. Results from large samples represent society, not individuals. Within this limit, it is important to note that all analyses used the whole sample (i.e., including adolescents with zero reported symptoms). Our assessment of problematic gaming was done on cross-sectional data, even though Internet Gaming Disorder is characterized by the 12-month *persistence* of symptoms, not their mere appearance during the period. For this reason, we do not present the “prevalence of IGD risk” specifically; the cross-sectional IGDS results would likely overestimate such risk. Finally, despite the evidential support for the IGDS psychometric properties, it should be noted that the IGDS has incomplete coverage of DSM-5 (e.g., continued use) and ICD-11 criteria (e.g., increasing priority and impairment-education) (King et al., 2020). Future research should assess the criterion and construct validity of the scale. Despite these limitations, by using large-scale representative samples of European adolescents, this study brought important insight into the prevalence of symptoms and gender, age, and cross-national comparability of the IGDS.

## Supplementary material

## Data Availability

Data are available upon request at hbsc.org. The analytical script is available at osf.io.
