# Supplementary material for: Internet gaming disorder scale: A comparison of symptoms prevalence, structure, and invariance in 12 nationally representative European adolescent samples
Source: J Behav Addict. 2025 Nov 5;14(4):1533–45. doi: 10.1556/2006.2025.00090 (PMC12767597; doi:10.1556/2006.2025.00090)
Supplement: Supplementary file 1 [file jba-14-1533-s001.pdf]

https://doi.org/10.1556/2006.2025.00090

Supplementary Material

Content

Table S1. *Number of missing items on the IGDS per region.*

Table S2. *The wording of the IGDS.*

Table S3. *Prevalence of individual IGD symptoms per gender, region, and age.*

Table S4. *Point-biserial correlation coefficients of the symptoms in girls (lower triangle) and boys (upper triangle).*

Table S5. *The goodness of fit of all factor models.*

Table S6. *Estimated dynamic cut-offs for each factor model.*

Table S7. *Measurement Invariance of the Main Groups*.

Table S8. *Measurement Invariance of the Region Groups*.

Figure S1. *Distribution of symptom prevalence per gender and region.*

Figure S2. *Network plots of IGDS per gender and region.*

Figure S3. *Network tree partitioning*.

Figure S4. *Stability of the edges of individual IGD symptoms – Boys, Cyprus*

Figure S5. *Stability of the edges of individual IGD symptoms – Girls, Cyprus*

Figure S6. *Stability of the edges of individual IGD symptoms – Boys, Czechia*

Figure S7. *Stability of the edges of individual IGD symptoms – Girls, Czechia*

Figure S8. *Stability of the edges of individual IGD symptoms – Boys, England*

Figure S9. *Stability of the edges of individual IGD symptoms – Girls, England*

Figure S10. *Stability of the edges of individual IGD symptoms – Boys, Estonia*

Figure S11. *Stability of the edges of individual IGD symptoms – Girls, Estonia*

Figure S12. *Stability of the edges of individual IGD symptoms – Boys, Iceland*

Figure S13. *Stability of the edges of individual IGD symptoms – Girls, Iceland*

Figure S14. *Stability of the edges of individual IGD symptoms – Boys, Malta*

Figure S15. *Stability of the edges of individual IGD symptoms – Girls, Malta*

Figure S16. *Stability of the edges of individual IGD symptoms – Boys, Netherlands*

Figure S17. *Stability of the edges of individual IGD symptoms – Girls, Netherlands*

Figure S18. *Stability of the edges of individual IGD symptoms – Boys, North Macedonia*

Figure S19. *Stability of the edges of individual IGD symptoms – Girls, North Macedonia*

Figure S20. *Stability of the edges of individual IGD symptoms – Boys, Scotland*

Figure S21. *Stability of the edges of individual IGD symptoms – Girls, Scotland*

Figure S22. *Stability of the edges of individual IGD symptoms – Boys, Serbia*

Figure S23. *Stability of the edges of individual IGD symptoms – Girls, Serbia*

Figure S24. *Stability of the edges of individual IGD symptoms – Boys, Slovakia*

Figure S25. *Stability of the edges of individual IGD symptoms – Girls, Slovakia*

Figure S26. *Stability of the edges of individual IGD symptoms – Boys, Slovenia*

Figure S27. *Stability of the edges of individual IGD symptoms – Girls, Slovenia*

Table S1. *Number of missing items on the IGDS per region.*

Dataset	Region	How many items did the respondent miss										Total
		**0**	**1**	**2**	**3**	**4**	**5**	**6**	**7**	**8**	**9**	
Official HBSC data from 12 regions that used IGDS.	CY	4247	167	21	14	7	3	3	4	15	186	4667
	CZ	9036	265	29	20	12	11	18	16	21	3478	12906
	EE	3667	48	5	3	5	1	4	1	8	1120	4862
	ENG	3052	80	9	6	2	9	8	7	8	1060	4241
	IS	1847	39	10	3	7	3	7	4	17	7961	9898
	MK	4006	35	9	0	0	2	0	1	1	7	4061
	MT	2953	100	11	2	3	5	2	4	6	321	3407
	NL	3509	19	3	1	2	1	0	1	2	778	4316
	RS	2313	94	17	13	8	7	5	6	14	1236	3713
	SCT	2142	26	4	2	19	42	3	6	12	2098	4354
	SI	4503	128	24	5	7	5	9	3	12	1631	6327
	SK	1587	101	20	9	9	10	7	8	14	3819	5584
	Total	42862	1102	162	78	81	99	66	61	130	23695	68336
Data after excluding cases with more than three missing IGDS items.	CY	4247	167	21	0	0	0	0	0	0	0	4435
	CZ	9036	265	29	0	0	0	0	0	0	0	9330
	EE	3667	48	5	0	0	0	0	0	0	0	3720
	ENG	3052	80	9	0	0	0	0	0	0	0	3141
	IS	1847	39	10	0	0	0	0	0	0	0	1896
	MK	4006	35	9	0	0	0	0	0	0	0	4050
	MT	2953	100	11	0	0	0	0	0	0	0	3064
	NL	3509	19	3	0	0	0	0	0	0	0	3531
	RS	2313	94	17	0	0	0	0	0	0	0	2424
	SCT	2142	26	4	0	0	0	0	0	0	0	2172
	SI	4503	128	24	0	0	0	0	0	0	0	4655
	SK	1587	101	20	0	0	0	0	0	0	0	1708
	Total	42862	1102	162	0	0	0	0	0	0	0	44126
Data after excluding cases with gender item missing.	CY	4226	167	21	0	0	0	0	0	0	0	4414
	CZ	9036	265	29	0	0	0	0	0	0	0	9330
	EE	3637	48	5	0	0	0	0	0	0	0	3690
	ENG	3035	79	9	0	0	0	0	0	0	0	3123
	IS	1778	37	8	0	0	0	0	0	0	0	1823
	MK	4003	35	9	0	0	0	0	0	0	0	4047
	MT	2923	99	11	0	0	0	0	0	0	0	3033
	NL	3509	19	3	0	0	0	0	0	0	0	3531
	RS	2313	94	17	0	0	0	0	0	0	0	2424
	SCT	2116	23	4	0	0	0	0	0	0	0	2143
	SI	4500	128	24	0	0	0	0	0	0	0	4652
	SK	1587	101	20	0	0	0	0	0	0	0	1708
	Total	42663	1095	160	0	0	0	0	0	0	0	43918

Note. In Czechia, England, Estonia, Scotland, Slovakia, and Slovenia, adolescents who responded “Never or almost never” to the filter question “How often do you play games” automatically skipped all IGDS items, thus resulting in nine items missed. In Iceland, the gaming-related questions were given to only a subset of the sample. North Macedonia did not ask “How often do you play games” and “On a day that you play games, how much time do you spend gaming”.

Table S2. *The wording of the IGDS.*

Symptom	Wording
	During the past year …
Preoccupation	have there been periods when all you could think of was the moment that you could play a game?
Tolerance	have you felt unsatisfied because you wanted to play more?
Withdrawal	have you been feeling miserable when you were unable to play a game?
Persistence	were you unable to reduce your time playing games after others had repeatedly told you to play less?
Escape	have you played games so that you would not have to think about annoying things?
Problems	have you had arguments with others about the consequences of your gaming behavior?
Deception	have you hidden the time you spend on games from others?
Displacement	have you lost interest in hobbies or other activities because gaming is all you wanted to do?
Conflict	have you experienced serious conflict with family or friends because of gaming?

Note. The response options were Yes and No.

Table S3. *Symptoms prevalences and differences in age.*

Region	Age/Test	Boys										Girls									
		n	Preoccupation	Tolerance	Withdrawal	Persistence	Escape	Problems	Deception	Displacement	Conflict	*n*	Preoccupation	Tolerance	Withdrawal	Persistence	Escape	Problems	Deception	Displacement	Conflict
Cyprus	11yo	662	45.2	35.1	32.0	29.9	47.9	24.2	17.8	12.7	14.6	678	27.6	26.1	21.2	22.5	38.2	14.3	10.7	9.1	9.1
	13yo	816	46.0	35.6	28.0	24.5	45.9	27.3	18.6	16.0	17.5	854	22.6	23.1	15.4	14.8	38.0	14.0	11.4	11.2	8.7
	15yo	676	38.6	33.1	21.3	19.9	40.9	22.2	15.3	14.0	15.7	719	17.3	17.7	13.0	12.4	28.8	10.1	6.6	7.3	6.3
	χ^2(Cramer *V*)		9.37**(0.07)	1.1 (0.02)	19.67***(0.1)	17.85***(0.09)	7.03*(0.06)	5.22 (0.05)	3.02 (0.04)	3.28 (0.04)	2.42 (0.03)		21.12***(0.1)	14.71***(0.08)	18.21***(0.09)	27.86***(0.11)	18.27***(0.09)	7.35*(0.06)	11.65**(0.07)	7.04*(0.06)	4.57 (0.05)
Czechia	11yo	1574	35.5	22.9	20.3	28.2	45.6	21.1	16.7	12.3	12.7	1479	18.2	12.1	9.9	24.5	42.3	10.0	11.6	7.5	8.6
	13yo	1902	39.6	26.7	19.4	22.6	52.0	22.7	16.3	12.1	14.5	1375	20.8	13.7	10.8	17.8	50.1	10.7	9.9	8.0	9.2
	15yo	1848	38.3	23.2	15.4	15.2	51.3	20.3	11.2	10.7	11.9	1152	18.3	13.6	7.5	7.8	45.4	6.8	6.1	7.4	5.1
	χ^2(Cramer *V*)		6.34*(0.03)	8.76*(0.04)	16.19***(0.06)	86.18***(0.13)	16.58***(0.06)	3.59 (0.03)	27.08***(0.07)	2.69 (0.02)	5.67 (0.03)		3.92 (0.03)	1.81 (0.02)	8.34*(0.05)	124.97***(0.18)	17.65***(0.07)	12.57**(0.06)	23.94***(0.08)	0.47 (0.01)	16.5***(0.06)
England	11yo	603	59.6	48.3	36.7	30.9	56.8	27.5	19.0	18.4	16.3	569	38.0	34.6	26.8	30.3	56.5	17.3	18.6	12.7	10.8
	13yo	635	54.9	46.8	31.4	28.5	51.9	25.6	17.9	15.1	14.0	545	29.5	34.0	17.7	25.0	50.8	12.5	15.1	12.3	10.8
	15yo	436	52.7	51.8	22.5	25.0	55.3	24.4	13.6	15.2	12.2	322	27.0	35.8	20.2	22.7	56.1	8.4	15.0	12.7	7.5
	χ^2(Cramer *V*)		5.38 (0.06)	2.63 (0.04)	23.83***(0.12)	4.37 (0.05)	3.08 (0.04)	1.36 (0.03)	5.55 (0.06)	2.9 (0.04)	3.58 (0.05)		14.34***(0.1)	0.3 (0.01)	14.21***(0.1)	7.3*(0.07)	4.16 (0.05)	14.62***(0.1)	3.09 (0.05)	0.04 (0.01)	3.13 (0.05)
Estonia	11yo	663	40.6	37.4	30.2	28.5	43.5	24.1	21.2	14.7	16.4	606	27.2	28.6	20.5	23.1	35.9	13.9	14.6	7.6	9.4
	13yo	776	42.6	39.9	28.5	24.0	46.0	22.6	19.1	13.3	14.8	542	29.0	25.9	24.8	20.2	41.0	16.1	15.9	8.9	10.4
	15yo	717	45.7	41.0	24.1	25.7	55.7	25.1	15.8	12.2	16.8	367	25.9	25.2	22.7	13.6	47.1	7.4	8.7	6.3	9.6
	χ^2(Cramer *V*)		3.8 (0.04)	1.93 (0.03)	6.8*(0.06)	3.9 (0.04)	23.25***(0.1)	1.39 (0.03)	6.83*(0.06)	1.93 (0.03)	1.2 (0.02)		1.09 (0.03)	1.75 (0.03)	3.05 (0.04)	13.13**(0.09)	12.17**(0.09)	15.14***(0.1)	10.24**(0.08)	2.11 (0.04)	0.31 (0.01)
Iceland	11yo	388	36.2	39.4	21.1	40.8	45.9	18.1	17.6	10.6	14.0	336	12.2	20.5	8.4	28.3	29.5	8.1	7.8	6.0	4.5
	13yo	349	37.0	37.0	18.3	25.8	41.1	18.1	16.0	11.2	12.9	243	10.7	16.0	6.6	19.3	23.0	6.2	4.9	5.3	5.4
	15yo	300	29.7	25.7	14.0	23.1	35.3	15.7	13.1	14.7	11.7	206	9.8	10.7	4.9	4.4	18.0	3.4	5.3	2.9	3.4
	χ^2(Cramer *V*)		4.51 (0.07)	15.44***(0.12)	5.82 (0.07)	30.82***(0.17)	7.72*(0.09)	0.82 (0.03)	2.62 (0.05)	3.04 (0.05)	0.73 (0.03)		0.8 (0.03)	9.04*(0.11)	2.49 (0.06)	46.7***(0.24)	9.41**(0.11)	4.8 (0.08)	2.41 (0.06)	2.59 (0.06)	0.97 (0.04)
Malta	11yo	609	53.6	43.6	32.4	36.8	58.1	27.6	17.5	13.5	14.6	611	40.1	37.6	29.3	32.6	55.8	20.7	15.6	13.7	10.3
	13yo	607	54.5	50.6	30.3	33.2	58.7	33.7	19.0	15.7	18.2	502	34.1	37.7	20.4	24.7	53.9	20.2	14.0	12.4	12.4
	15yo	385	54.8	51.3	24.7	31.8	62.5	35.1	24.7	16.4	21.1	319	24.2	28.9	13.5	20.4	46.7	11.3	10.0	10.4	8.5
	χ^2(Cramer *V*)		0.15 (0.01)	8.03*(0.07)	6.74*(0.07)	3.18 (0.04)	2.07 (0.04)	7.78*(0.07)	7.9*(0.07)	1.87 (0.03)	7.01*(0.07)		23.3***(0.13)	8.2*(0.08)	32.08***(0.15)	17.77***(0.11)	7.2*(0.07)	13.86***(0.1)	5.48 (0.06)	2.05 (0.04)	3.28 (0.05)
Netherlands	11yo	776	31.2	17.5	19.7	16.9	43.7	13.3	7.9	8.1	3.6	661	12.0	7.9	9.1	8.0	37.4	5.5	7.7	3.2	1.1
	13yo	725	28.8	24.8	29.6	17.8	45.5	21.5	12.1	11.0	6.5	461	15.0	10.8	14.1	8.7	34.9	9.3	7.8	6.1	3.7
	15yo	578	24.2	18.9	21.5	10.7	42.7	17.0	9.0	9.2	4.5	317	12.9	8.5	10.1	5.7	35.3	6.3	9.1	6.6	3.2
	χ^2(Cramer *V*)		8.06*(0.06)	13.52**(0.08)	22.36***(0.1)	14.16***(0.08)	1.08 (0.02)	17.76***(0.09)	8.26*(0.06)	3.74 (0.04)	6.82*(0.06)		2.15 (0.04)	3.05 (0.05)	7.28*(0.07)	2.54 (0.04)	0.86 (0.02)	6.53*(0.07)	0.64 (0.02)	7.61*(0.07)	9.19*(0.08)
North Macedonia	11yo	573	57.4	41.7	38.7	36.5	50.9	26.6	23.4	25.1	20.7	647	41.6	29.2	26.0	30.0	47.4	15.5	14.8	14.1	13.0
	13yo	558	59.0	40.7	35.5	36.3	58.0	20.9	19.0	17.4	16.5	663	35.5	29.3	17.9	22.6	49.8	12.5	11.2	12.1	11.9
	15yo	801	58.0	39.1	29.3	33.4	56.0	22.9	16.9	17.9	18.2	803	27.4	23.2	16.1	19.2	46.9	13.6	10.3	13.3	10.0
	χ^2(Cramer *V*)		0.3 (0.01)	0.94 (0.02)	13.9***(0.09)	1.89 (0.03)	6.27*(0.06)	5.33 (0.05)	9.29**(0.07)	13.97***(0.09)	3.32 (0.04)		32.61***(0.12)	9.23**(0.07)	24.11***(0.11)	23.89***(0.11)	1.39 (0.03)	2.41 (0.03)	7.43*(0.06)	1.17 (0.02)	3.41 (0.04)
Scotland	11yo	0	-	-	-	-	-	-	-	-	-	0	-	-	-	-	-	-	-	-	-
	13yo	696	59.3	41.2	21.9	26.3	59.6	28.9	13.8	11.7	11.4	585	30.1	25.3	13.8	19.9	46.0	11.1	12.0	9.8	7.1
	15yo	498	55.4	43.8	21.3	28.7	63.2	29.4	15.1	13.5	13.7	355	26.2	22.5	15.8	17.5	41.1	9.6	8.8	6.5	4.2
	χ^2(Cramer *V*)		1.9 (0.04)	2.94 (0.05)	0.3 (0.02)	0.88 (0.03)	3.5 (0.05)	0.06 (0.01)	0.9 (0.03)	1.29 (0.03)	1.88 (0.04)		4.16 (0.07)	4.03 (0.07)	6.48*(0.08)	5.1 (0.07)	3.38 (0.06)	8.97*(0.1)	10.57**(0.11)	13.65**(0.12)	18.5***(0.14)
Serbia	11yo	419	43.9	31.9	23.7	25.2	45.4	18.2	21.8	16.5	14.9	343	21.0	13.5	8.8	16.2	30.1	11.5	8.8	8.5	6.7
	13yo	457	42.6	30.0	22.2	25.1	49.1	18.3	17.0	17.1	14.3	337	21.4	17.0	11.1	17.0	43.4	8.4	11.9	11.0	6.5
	15yo	458	42.1	27.4	14.4	19.7	44.1	15.6	16.2	14.9	12.1	392	25.0	15.8	10.0	14.5	39.1	8.4	9.5	11.5	7.1
	χ^2(Cramer *V*)		0.29 (0.01)	2.13 (0.04)	13.87***(0.1)	4.93 (0.06)	2.46 (0.04)	1.43 (0.03)	5.33 (0.06)	0.84 (0.03)	1.63 (0.04)		2.06 (0.04)	1.72 (0.04)	0.93 (0.03)	0.85 (0.03)	13.25**(0.11)	2.64 (0.05)	2 (0.04)	1.99 (0.04)	0.11 (0.01)
Slovakia	11yo	0	-	-	-	-	-	-	-	-	-	0	-	-	-	-	-	-	-	-	-
	13yo	475	54.9	32.0	33.7	26.7	53.3	27.4	24.9	13.4	16.6	416	35.2	21.9	18.3	25.1	40.1	12.5	15.3	8.2	9.4
	15yo	516	50.5	30.0	29.7	24.4	54.7	28.2	18.6	14.9	17.6	297	27.7	18.0	14.5	13.0	43.3	7.2	8.1	7.8	7.4
	χ^2(Cramer *V*)		1.92 (0.04)	0.79 (0.03)	2.07 (0.05)	1.28 (0.04)	0.21 (0.01)	0.57 (0.02)	6.86*(0.08)	2.54 (0.05)	1.68 (0.04)		4.73 (0.08)	2.75 (0.06)	3.4 (0.07)	16.8***(0.15)	0.78 (0.03)	8.38*(0.11)	10.7**(0.12)	4.73 (0.08)	5.19 (0.09)
Slovenia	11yo	875	33.0	20.8	20.8	21.0	27.6	15.4	16.9	11.2	10.8	803	18.8	12.7	12.5	13.0	25.0	9.4	10.9	8.4	6.4
	13yo	870	42.5	26.5	23.1	20.1	35.2	19.3	16.4	13.4	12.9	666	21.5	14.4	13.1	12.3	30.7	12.8	12.6	9.8	7.4
	15yo	853	41.1	25.6	20.4	19.3	38.1	19.7	19.0	14.1	14.2	560	20.6	14.7	11.3	13.3	33.7	9.7	9.7	10.8	7.2
	χ^2(Cramer *V*)		19.3***(0.09)	8.89*(0.06)	2.18 (0.03)	0.75 (0.02)	22.5***(0.09)	6.44*(0.05)	2.25 (0.03)	3.52 (0.04)	4.54 (0.04)		1.73 (0.03)	1.39 (0.03)	0.94 (0.02)	0.27 (0.01)	12.99**(0.08)	5.23 (0.05)	2.74 (0.04)	2.19 (0.03)	0.64 (0.02)

Note. Scotland and Slovakia did not collect data from 11-year-olds. The gender samples in the table excluded cases that did not respond to the gender item.

$^{*}p < 0.05$. $^{**}p < 0.01$. $^{***}p < 0.001$.

The largest differences (Cramer V > 0.15) in age groups were in Persistence: data showed a steep decline in the presence of this symptom as adolescents were older in Czech (24.5 % → 17.8 % → 7.8 %) and Slovakian girls (17.8 % → 7.8 %) and both Icelandic girls (28.3 % → 19.3 % → 4.4 %) and boys (40.8 % → 25.8 % → 23.1 %). Moderate differences (0.10 ≤ *V* < 0.15) in this symptom were also found in Maltese, Cyprian, and North Macedonian girls and Czech boys. Besides this symptom, Withdrawal also tended to decrease moderately with older age, especially in Malta, England, North Macedonia, the Netherlands, and Serbia. Non-significant age differences were primarily found in Displacement, Conflict, and Preoccupation.

Table S4. *Point-biserial correlation coefficients of the symptoms in girls (lower triangle) and boys (upper triangle).*

	1	2	3	4	5	6	7	8	9
1. Preoccupation	1	0.593	0.596	0.450	0.501	0.482	0.485	0.471	0.482
2. Tolerance	0.675	1	0.664	0.513	0.431	0.554	0.529	0.567	0.534
3. Withdrawal	0.674	0.710	1	0.499	0.459	0.538	0.582	0.580	0.603
4. Persistence	0.539	0.545	0.540	1	0.394	0.522	0.540	0.576	0.538
5. Escape	0.546	0.524	0.522	0.454	1	0.431	0.459	0.369	0.448
6. Problems	0.561	0.594	0.586	0.586	0.486	1	0.583	0.625	0.672
7. Deception	0.556	0.572	0.607	0.568	0.516	0.649	1	0.622	0.674
8. Displacement	0.575	0.623	0.614	0.606	0.464	0.684	0.635	1	0.656
9. Conflict	0.561	0.564	0.616	0.561	0.488	0.754	0.678	0.668	1

Note. Girls' data are in the lower triangle and boys' data are in the upper triangle. All correlations are significant on the 0.05 alpha level. The gender samples in the table excluded cases that did not respond to the gender item.

Table S5. *The goodness of fit of all factor models.*

Goodness-of-fit indices of all models.

Sample	*n*	*# of parameters*	*df*	χ^2	*CFI*	*TLI*	*SRMR*	*RMSEA*	*RMSEA 90% CI*		*level of misspecification*[1]
									lower	*upper*	
Full sample	44,126	18	27	4810.478	0.946	0.928	0.035	0.064	0.063	0.066	moderate
Gender											
Boys	24,149	18	27	2605.246	0.944	0.925	0.036	0.064	0.062	0.066	moderate
Girls	19,769	18	27	2371.017	0.941	0.921	0.037	0.067	0.065	0.069	moderate
Region											
Cyprus	4,435	18	27	470.842	0.952	0.936	0.034	0.062	0.057	0.067	moderate
Czechia	9,330	18	27	1090.111	0.937	0.916	0.037	0.066	0.063	0.069	moderate
Estonia	3,720	18	27	409.588	0.949	0.932	0.035	0.062	0.057	0.068	moderate
England	3,141	18	27	532.234	0.916	0.888	0.046	0.078	0.073	0.084	high
Scotland	2,172	18	27	474.85	0.888	0.851	0.052	0.088	0.081	0.095	high
Iceland	1,896	18	27	201.169	0.96	0.947	0.033	0.059	0.052	0.067	moderate
Macedonia	4,050	18	27	615.207	0.921	0.894	0.043	0.074	0.069	0.079	high
Malta	3,064	18	27	345.03	0.937	0.916	0.038	0.063	0.057	0.069	moderate
Netherlands	3,531	18	27	339.075	0.941	0.922	0.035	0.057	0.052	0.063	moderate
Serbia	2,424	18	27	305.198	0.953	0.938	0.036	0.067	0.06	0.074	moderate
Slovenia	4,655	18	27	550.92	0.96	0.946	0.032	0.066	0.061	0.07	moderate
Slovakia	1,708	18	27	354.741	0.918	0.89	0.046	0.087	0.079	0.096	high
Gender * Region											
Boys											
Cyprus	2,162	18	27	288.292	0.937	0.915	0.039	0.069	0.061	0.076	moderate
Czechia	5,324	18	27	655.967	0.932	0.909	0.039	0.067	0.063	0.072	moderate
Estonia	2,166	18	27	269.166	0.946	0.928	0.037	0.065	0.058	0.072	moderate
England	1,683	18	27	273.109	0.92	0.894	0.045	0.075	0.067	0.083	high
Scotland	1,200	18	27	255.425	0.879	0.839	0.053	0.085	0.075	0.094	high
Iceland	1,038	18	27	107.602	0.964	0.952	0.033	0.054	0.044	0.065	moderate
Macedonia	1,934	18	27	295.932	0.916	0.888	0.044	0.072	0.065	0.08	high
Malta	1,601	18	27	167.134	0.936	0.915	0.037	0.058	0.05	0.067	moderate
Netherlands	2,089	18	27	213.871	0.94	0.92	0.036	0.058	0.051	0.065	moderate
Serbia	1,346	18	27	151.891	0.959	0.946	0.036	0.06	0.051	0.07	moderate
Slovenia	2,613	18	27	383.228	0.951	0.934	0.037	0.072	0.066	0.079	high
Slovakia	993	18	27	208.712	0.92	0.894	0.046	0.085	0.075	0.096	high
Girls											

Cyprus	2,252	18	27	205.811	0.963	0.95	0.032	0.055	0.048	0.062	moderate
Czechia	4,006	18	27	587.54	0.923	0.897	0.041	0.073	0.068	0.078	high
Estonia	1,524	18	27	182.37	0.942	0.923	0.036	0.062	0.054	0.071	moderate
England	1,440	18	27	332.613	0.894	0.859	0.052	0.09	0.081	0.099	high
Scotland	943	18	27	250.639	0.889	0.852	0.056	0.094	0.084	0.105	high
Iceland	785	18	27	192.617	0.896	0.862	0.052	0.09	0.078	0.102	high
Macedonia	2,113	18	27	344.493	0.919	0.892	0.044	0.075	0.068	0.082	high
Malta	1,432	18	27	261.238	0.917	0.889	0.047	0.079	0.071	0.088	high
Netherlands	1,442	18	27	145.47	0.938	0.917	0.037	0.055	0.047	0.064	moderate
Serbia	1,078	18	27	222.573	0.929	0.905	0.044	0.084	0.074	0.094	high
Slovenia	2,039	18	27	209.847	0.967	0.956	0.029	0.058	0.051	0.066	moderate
Slovakia	715	18	27	194.276	0.891	0.854	0.055	0.097	0.084	0.11	high

Note. df = degrees of freedom. CFI = Comparative Fit Index. TLI = Tucker-Lewis Index. SRMR = Standardized root mean squared residual. RMSEA = Root mean squared error of approximation. CI = Confidence interval. Full sample and Region samples included cases that did not indicated their gender. Gender and Gender*region samples excluded cases that did not respond to the gender item.

[1]. This evaluation is based on McNeish & Wolf (2023). We estimated custom dynamic fit cut-offs of the CFI, SRMR, and RMSEA for each model. Level-1 cut-offs represent ‘small’ misspecification, Level-2 cut-offs are a ‘moderate’ misspecification, and Level-3 cut-offs are a ‘large’ misspecification. When a model's fit indices reach different levels of misspecification, the average or most prevalent level is chosen (e.g., in the case of CFI - Level 1, SRMR - Level 2, and RMSEA - Level 2, we evaluate the misspecification as *moderate*.

Table S6. *Estimated dynamic cut-offs for each factor model.*

	CFI levels			RMSEA levels			SRMR levels		
Model	1	2	3	1	2	3	1	2	3
Region									
Cyprus	0.973	0.944	0.89	0.047	0.069	0.104	0.027	0.037	0.052
Czechia	0.965	0.934	0.874	0.049	0.07	0.104	0.032	0.041	0.056
Estonia	0.971	0.941	0.885	0.047	0.069	0.103	0.028	0.038	0.053
England	0.969	0.939	0.877	0.047	0.068	0.104	0.029	0.038	0.055
Scotland	0.966	0.931	0.87	0.047	0.07	0.104	0.029	0.04	0.056
Iceland	0.974	0.951	0.899	0.049	0.069	0.104	0.029	0.037	0.051
North Macedonia	0.965	0.933	0.873	0.048	0.07	0.103	0.031	0.04	0.055
Malta	0.965	0.931	0.865	0.047	0.069	0.104	0.03	0.04	0.057
Netherlands	0.963	0.922	0.853	0.046	0.07	0.104	0.03	0.041	0.058
Serbia	0.975	0.951	0.904	0.049	0.07	0.103	0.03	0.038	0.05
Slovenia	0.978	0.957	0.914	0.048	0.07	0.103	0.026	0.035	0.048
Slovakia	0.976	0.951	0.901	0.046	0.069	0.103	0.027	0.036	0.051
Gender * Region									
Boys									
Cyprus	0.971	0.938	0.88	0.047	0.07	0.104	0.028	0.039	0.054
Czechia	0.964	0.932	0.87	0.049	0.07	0.104	0.032	0.041	0.056
Estonia	0.97	0.943	0.887	0.048	0.069	0.103	0.029	0.038	0.053
England	0.97	0.938	0.876	0.045	0.068	0.103	0.028	0.038	0.055
Scotland	0.964	0.925	0.855	0.045	0.068	0.102	0.032	0.042	0.059
Iceland	0.975	0.949	0.899	0.046	0.067	0.1	0.03	0.039	0.052
North Macedonia	0.961	0.926	0.86	0.049	0.07	0.104	0.032	0.042	0.058
Malta	0.961	0.92	0.847	0.046	0.069	0.103	0.03	0.042	0.059
Netherlands	0.962	0.922	0.852	0.047	0.069	0.103	0.03	0.041	0.058
Serbia	0.975	0.952	0.903	0.047	0.068	0.102	0.031	0.039	0.051
Slovenia	0.978	0.956	0.912	0.048	0.07	0.103	0.027	0.035	0.048
Slovakia	0.978	0.955	0.903	0.045	0.065	0.101	0.029	0.037	0.051
Girls									
Cyprus	0.975	0.945	0.892	0.046	0.07	0.104	0.026	0.037	0.052
Czechia	0.966	0.933	0.871	0.049	0.07	0.104	0.031	0.04	0.056

Estonia	0.969	0.936	0.875	0.046	0.068	0.102	0.029	0.039	0.055
England	0.971	0.94	0.884	0.046	0.068	0.102	0.029	0.039	0.054
Scotland	0.975	0.946	0.89	0.043	0.066	0.1	0.029	0.039	0.053
Iceland	0.975	0.945	0.892	0.043	0.066	0.099	0.031	0.041	0.054
North Macedonia	0.966	0.933	0.873	0.048	0.07	0.104	0.03	0.041	0.055
Malta	0.971	0.943	0.883	0.046	0.068	0.102	0.029	0.039	0.054
Netherlands	0.96	0.916	0.843	0.045	0.068	0.101	0.03	0.042	0.059
Serbia	0.979	0.956	0.912	0.045	0.067	0.1	0.028	0.036	0.049
Slovenia	0.979	0.956	0.911	0.047	0.069	0.104	0.025	0.034	0.048
Slovakia	0.98	0.952	0.899	0.04	0.064	0.099	0.029	0.039	0.054

Note. CFI = Comparative Fit Index. TLI = Tucker-Lewis Index. RMSEA = Root mean squared error of approximation. We estimated each model's custom dynamic fit cut-offs of the CFI, SRMR, and RMSEA. Level-1 cut-offs represent a maximum of 'small' misspecification, Level-2 cut-offs are a maximum of 'moderate' misspecification, and Level-3 cut-offs are a maximum of 'large' misspecification.

Table S7. *Measurement Invariance of the Main Groups*.

Sample	Model	χ^2 (*df*)	*AIC*	*BIC*	*CFI*	*TLI*	*SRMR*	*RMSEA*	RMSEA 90% CI		$\Delta\chi^2$	Δdf	p ($\Delta\chi^2$)	*ΔCFI*	*ΔTLI*	*ΔRMSEA*
									Lower	Upper						
Full sample (n = 43,918)																
Across 2 genders	Configural	4976.263 (54)	281922.9	282390.6	*0.943*	*0.923*	0.033	*0.065*	0.064	0.067						
	Metric	5591.353 (63)	282519.9	282909.7	*0.935*	*0.926*	0.06	*0.064*	0.063	0.066	615.0891	9	*< 0.001*	−0.007	0.003	0.001
	Scalar	6565.613 (71)	283478.2	283798.7	*0.924*	*0.923*	0.063	*0.065*	0.064	0.067	974.2608	8	*< 0.001*	−0.011	−0.003	0.001
Boys (n = 24,149)																
Across 12 regions	Configural	3270.329 (324)	181345	183956.9	*0.936*	*0.915*	0.036	*0.068*	0.066	0.07						
	Metric	3902.257 (423)	181779	183592.8	*0.925*	*0.923*	0.067	*0.065*	0.063	0.067	631.93	99	*< 0.001*	−0.012	0.008	0.003
	Scalar	5835.011 (511)	183535.7	184640.1	*0.885*	*0.902*	0.075	*0.073*	0.071	0.075	1932.75	88	*< 0.001*	*−0.04*	*−0.021*	0.008
Girls (n = 19,769)																
Across 12 regions	Configural	3129.488 (324)	87575.18	90123.36	*0.929*	*0.906*	0.038	*0.073*	0.071	0.076						
	Metric	4029.181 (423)	88276.87	90046.44	*0.909*	*0.907*	0.089	*0.073*	0.071	0.075	899.69	99	*< 0.001*	−0.02	0.001	0.001
	Scalar	5362.871 (511)	89434.56	90512.03	*0.878*	*0.897*	0.094	*0.077*	0.075	0.079	1333.69	88	*< 0.001*	*−0.031*	−0.011	0.004

Note. AIC = Akaike information criterion. BIC = Bayesian information criterion. df = Degrees of freedom. CFI = Comparative Fit Index. TLI = Tucker-Lewis Index. SRMR = Standardized root mean squared residual. RMSEA = Root mean squared error of approximation. CI = Confidence interval. Parameters exceeding the set criteria for acceptable invariance level are in italics: ΔCFI and ΔTLI ≤ 0.02 and ΔRMSEA ≤ 0.03. The samples in the table excluded cases that did not respond to the gender item.

Table S8. *Measurement Invariance of the Region Groups.*

Sample	Model	χ^2 (*df*)	*AIC*	*BIC*	*CFI*	*TLI*	*SRMR*	*RMSEA*	RMSEA 90% CI		$\Delta\chi^2$	Δdf	p ($\Delta\chi^2$)	*ΔCFI*	*ΔTLI*	*ΔRMSEA*
									Lower	Upper						
Region Group 1 (Serbia, Iceland, Czechia, Slovenia, Netherlands)																
Boys (n = 12,410)																
Across 5 regions	Configural	1512.6 (135)	81862.212	82860.649	0.945	0.926	0.034	0.065	0.062	0.068						
	Metric	1918.3 (171)	82195.907	82928.094	0.93	0.926	0.074	0.065	0.063	0.068	405.69	36	*< 0.001*	−0.015	0	0
	Scalar	2666.6 (203)	82880.27	83375.79	0.901	0.912	0.08	0.071	0.069	0.073	748.36	32	*< 0.001*	*−0.029*	−0.014	0.006
Girls (n = 9,350)																
Across 5 regions	Configural	1358.047 (135)	25498.849	26459.484	0.936	0.914	0.036	0.071	0.067	0.074						
	Metric	1817.516 (171)	25886.318	26590.783	0.914	0.909	0.092	0.073	0.070	0.076	459.47	36	*< 0.001*	*−0.022*	−0.005	0.002
	Scalar	2357.384 (203)	26362.186	26838.945	0.887	0.9	0.097	0.076	0.074	0.079	539.87	32	*< 0.001*	*−0.027*	−0.009	0.004
Region Group 2a (Slovakia, Estonia, Cyprus)																
Boys (n = 5,321)																
Across 3 regions	Configural	766.17 (81)	42263.688	42793.486	0.937	0.916	0.036	0.07	0.066	0.075						
	Metric	793.34 (99)	42254.853	42666.918	0.936	0.93	0.042	0.064	0.060	0.068	27.165	18	0.076	−0.001	0.014	0.006
	Scalar	910.54 (115)	42340.056	42647.469	0.927	0.931	0.045	0.064	0.060	0.068	117.203	16	*< 0.001*	−0.009	0.001	0

Girls (n = 4,491)																	
	Across 3 regions	Configural	582.457 (81)	25498.849	26459.484	0.944	0.926	0.034	0.065	0.061	0.071						
		Metric	615.979 (91)	25886.318	26590.783	0.943	0.937	0.038	0.06	0.056	0.065	459.47	18	*< 0.001*	−0.002	0.012	0.005
		Scalar	706.438 (115)	26362.186	26838.945	0.934	0.938	0.041	0.06	0.056	0.064	539.87	16	*< 0.001*	−0.008	0.001	0
Region Group 2b (Malta, North Macedonia, England, Scotland)																	
Boys (n = 24,149)																	
	Across 4 regions	Configural	991.6 (108)	57219.124	57947.356	0.915	0.886	0.04	0.072	0.068	0.076						
		Metric	1091.5 (135)	57265.066	57811.24	0.908	0.902	0.052	0.067	0.064	0.071	99.942	27	*< 0.001*	−0.007	0.015	0.005
		Scalar	1374.7 (159)	57500.215	57884.56	0.883	0.894	0.058	0.07	0.067	0.073	283.149	24	*< 0.001*	*−0.025*	−0.008	0.003
Girls (n = 6,418)																	
	Across 4 regions	Configural	1188.984 (108)	39349.748	40069.838	0.907	0.876	0.045	0.083	0.079	0.087						
		Metric	1280.06 (135)	39386.823	39926.891	0.902	0.895	0.056	0.076	0.073	0.080	91.076	27	*< 0.001*	−0.006	0.019	0.007
		Scalar	1391.377 (159)	39450.14	39830.188	0.894	0.904	0.058	0.073	0.070	0.077	111.317	24	*< 0.001*	−0.008	0.009	0.003

Note. AIC = Akaike information criterion. BIC = Bayesian information criterion. df = Degrees of freedom. CFI = Comparative Fit Index. TLI = Tucker-Lewis Index. SRMR = Standardized root mean squared residual. RMSEA = Root mean squared error of approximation. CI = Confidence interval. Parameters exceeding the set criteria for acceptable invariance level are in italics: ΔCFI and ΔTLI ≤ 0.01 (all invariance levels) and ΔRMSEA ≤ 0.015 (all invariance levels). The gender samples in the table excluded cases that did not respond to the gender item.

Figure S1. *Distribution of symptom prevalence per gender and region.*

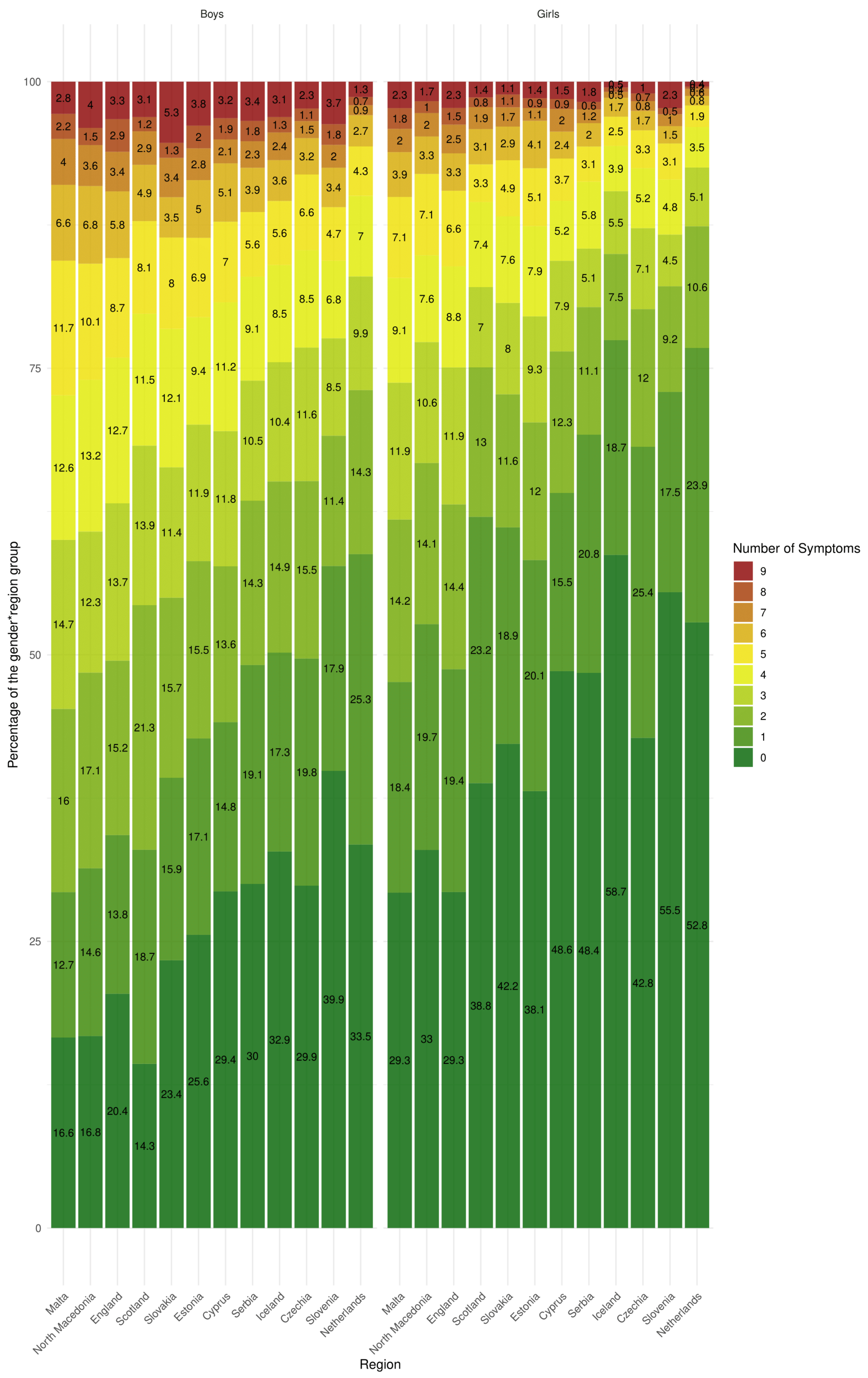

Note. The gender samples in the table excluded cases that did not respond to the gender item.

Figure S2. *Network plots of IGDS per gender and region.*

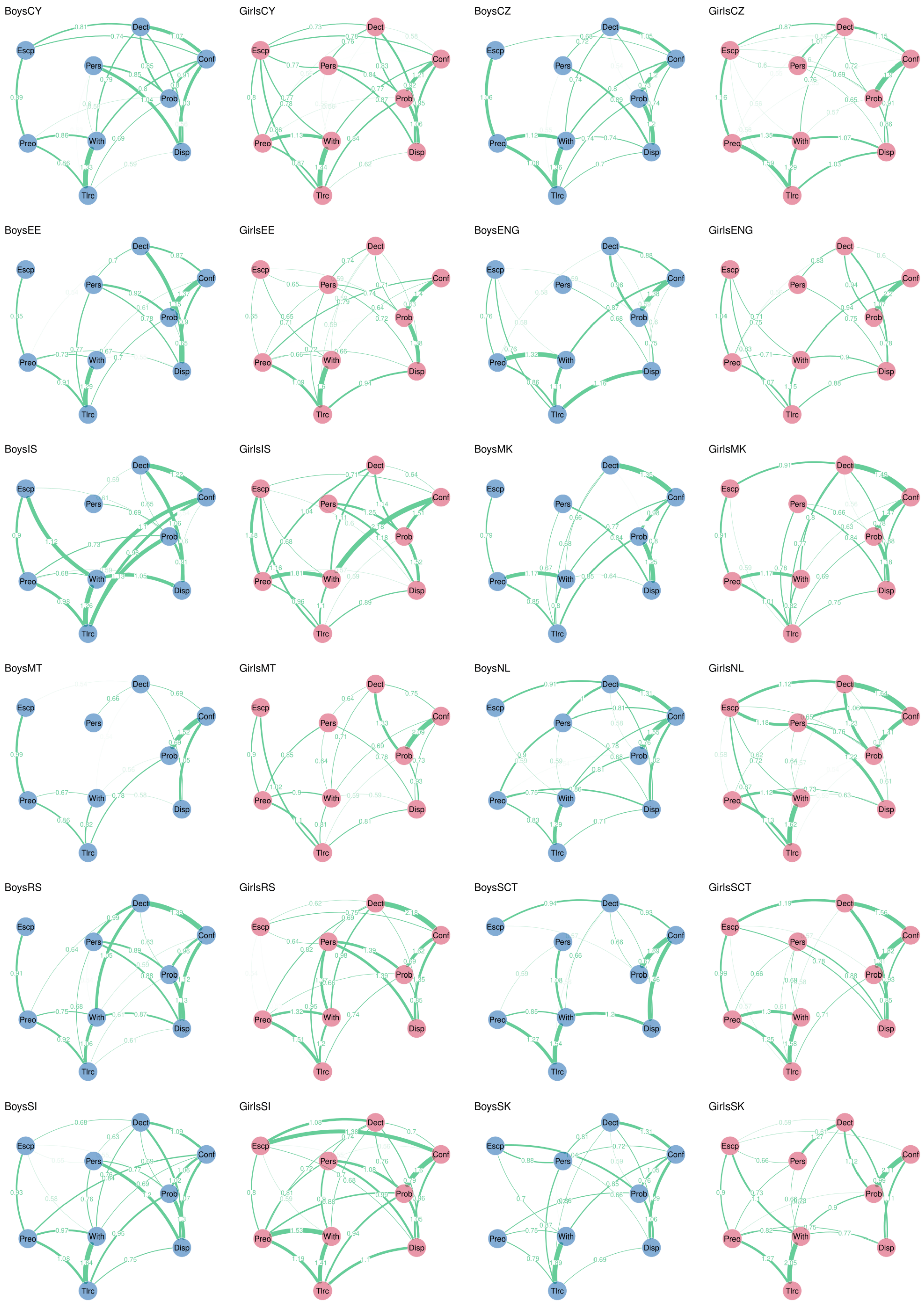

Figure S3. *Network tree partitioning*.

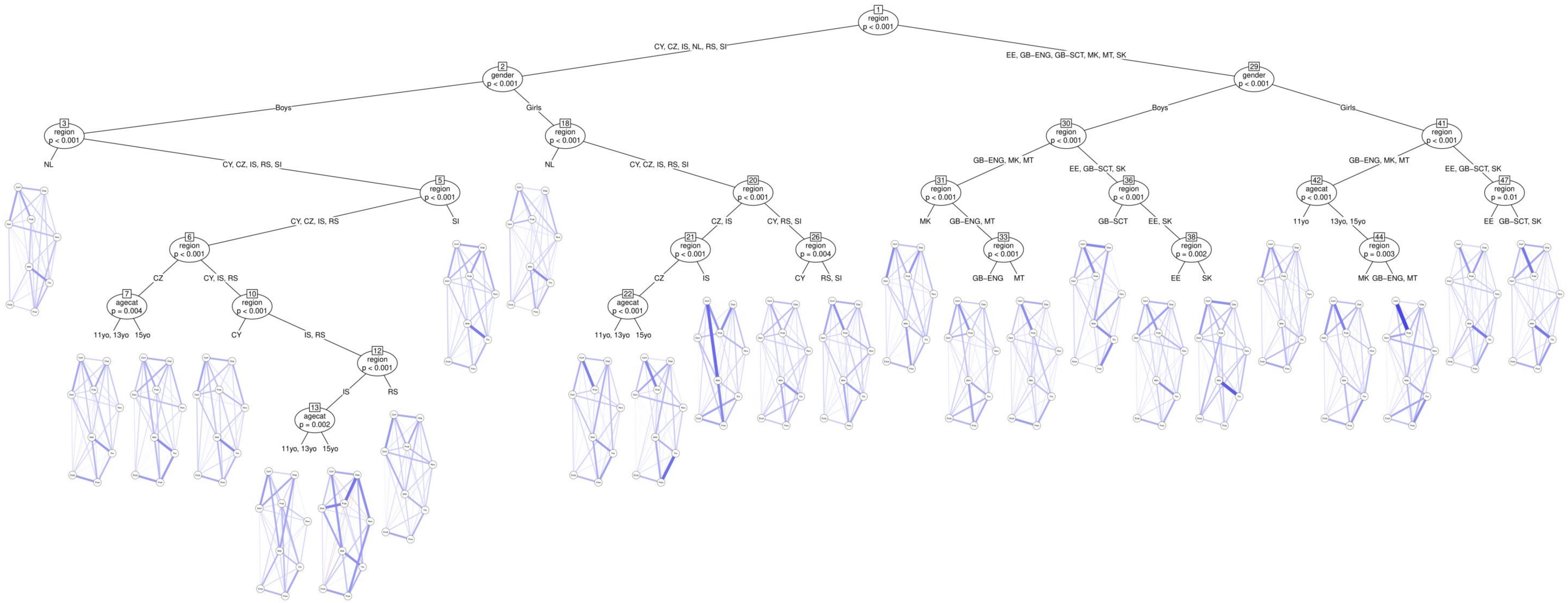

Figure S4. *Stability of the edges of individual IGD symptoms – Boys, Cyprus*

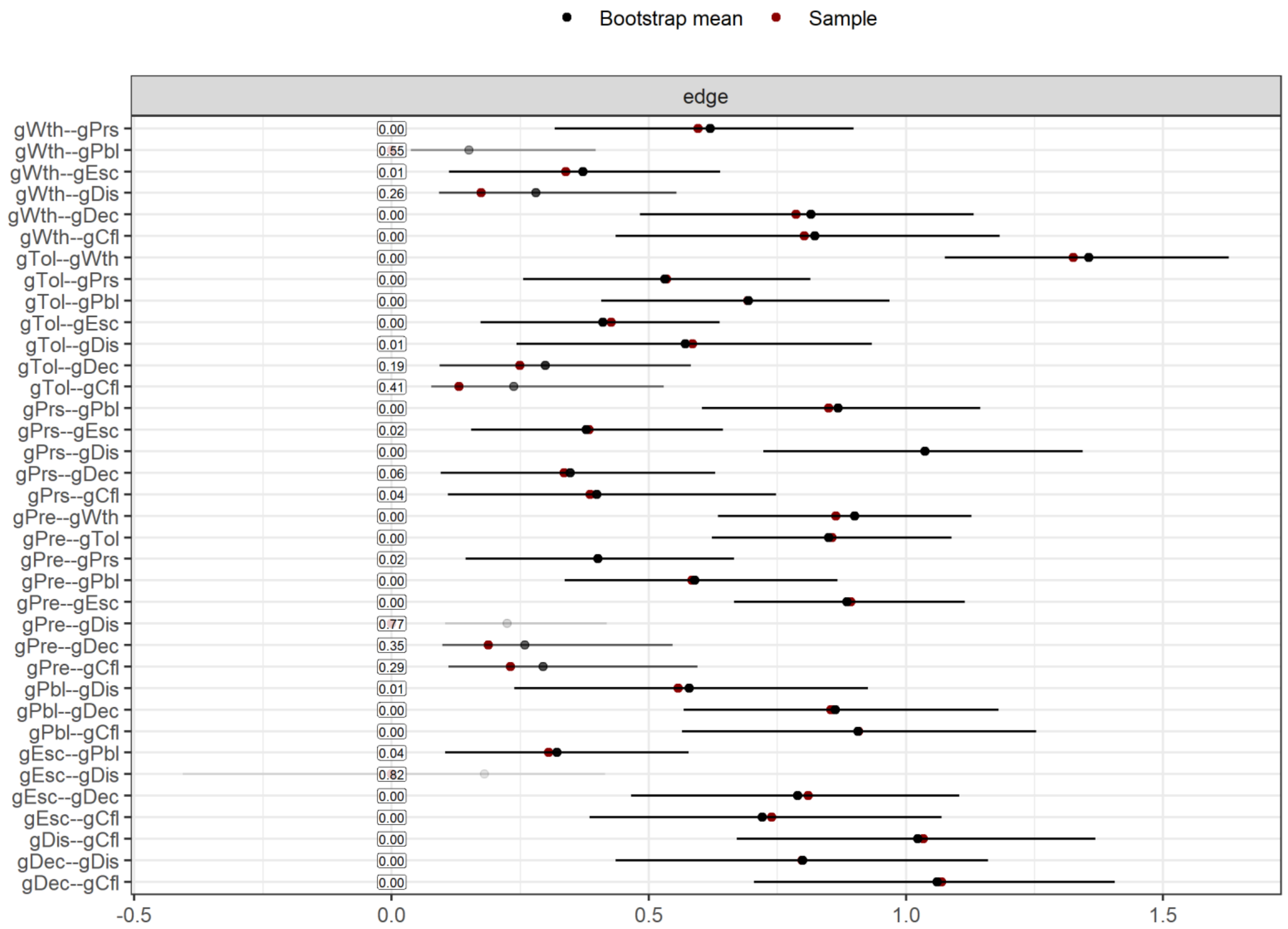

Note: The numbers in rectangles show how often an edge was estimated non-zero in the 1000 bootstraps.

Figure S5. *Stability of the edges of individual IGD symptoms – Girls, Cyprus*

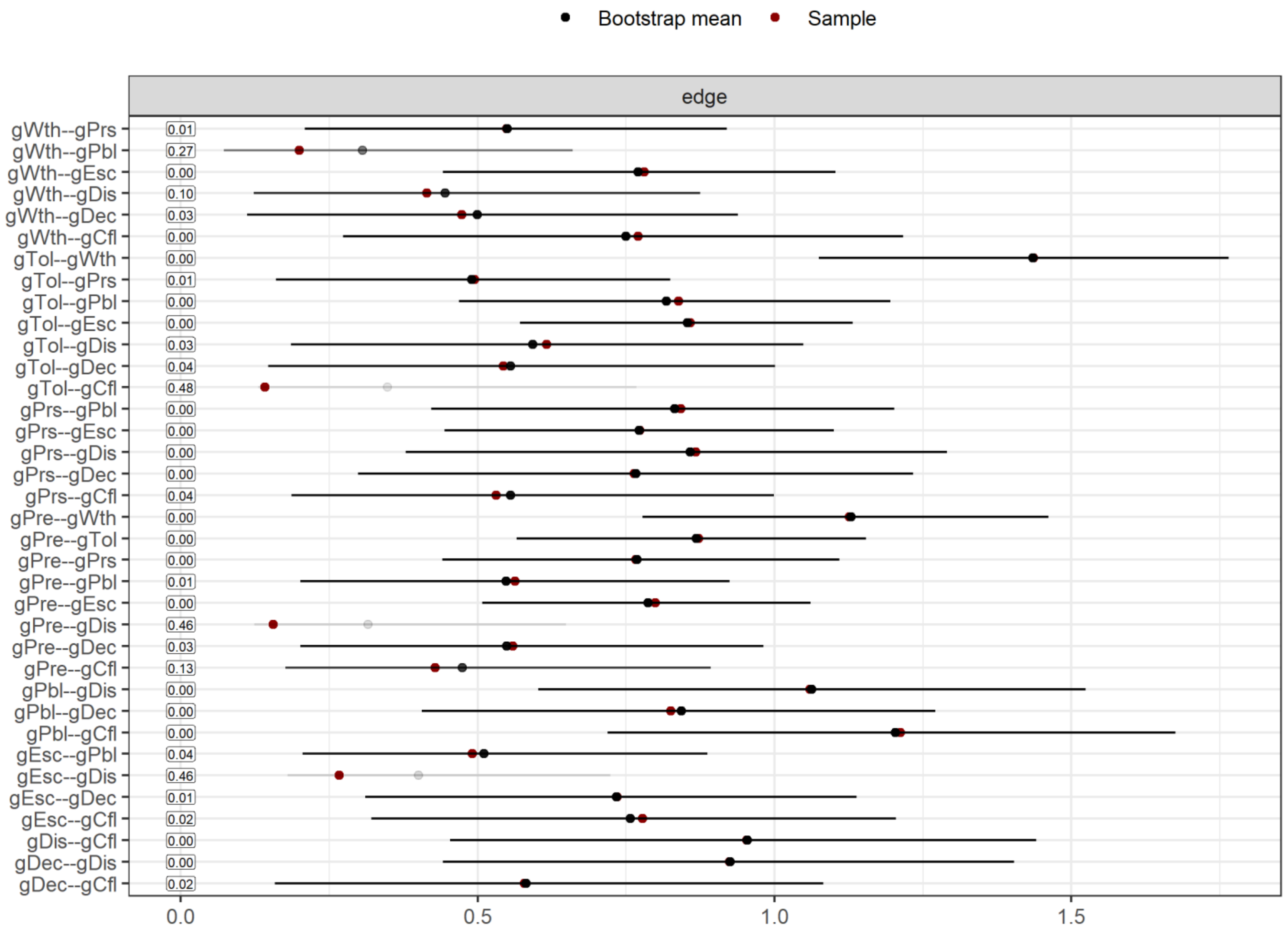

Note: The numbers in rectangles show how often an edge was estimated non-zero in the 1000 bootstraps.

Figure S6. *Stability of the edges of individual IGD symptoms – Boys, Czechia*

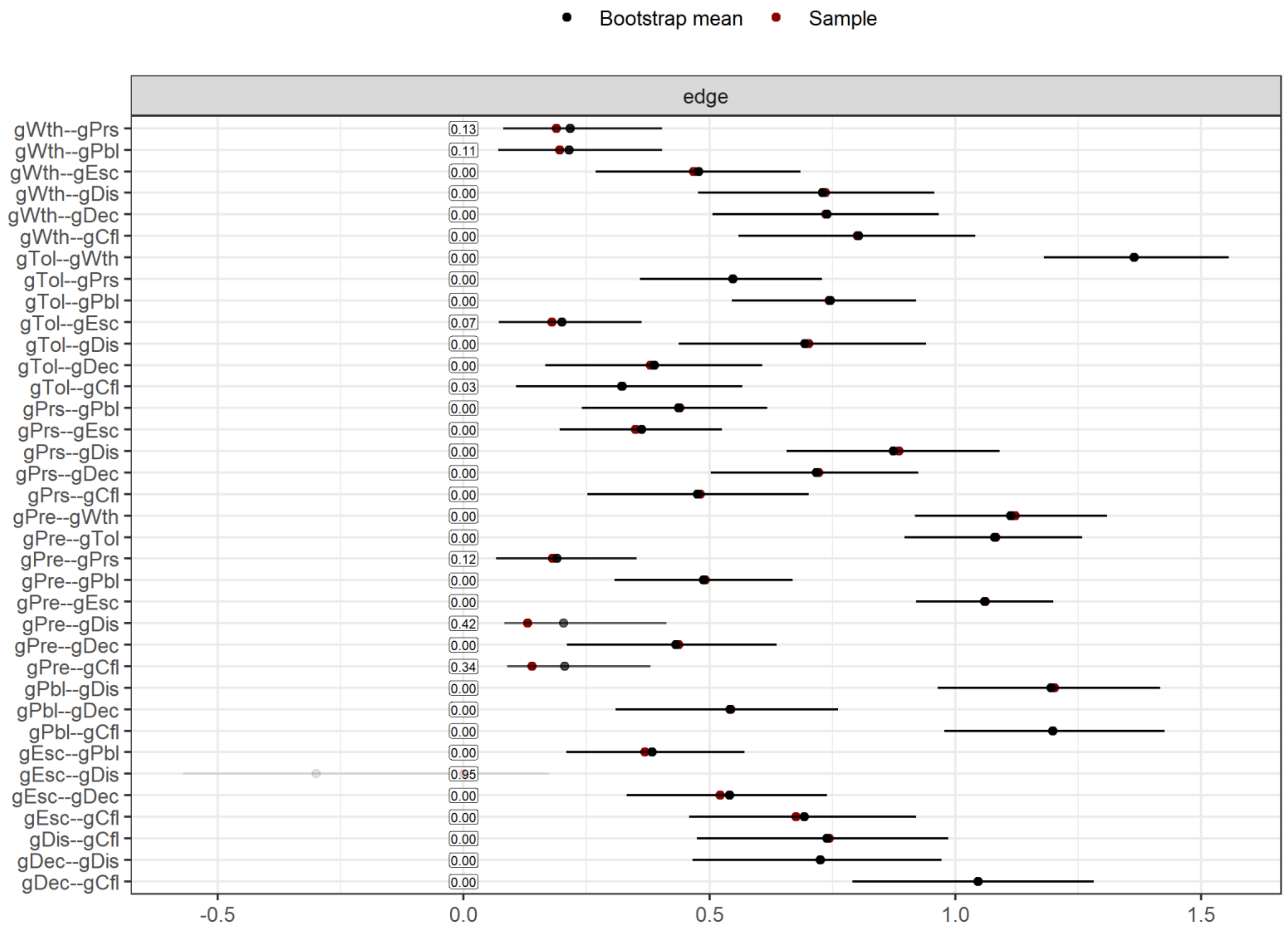

Note: The numbers in rectangles show how often an edge was estimated non-zero in the 1000 bootstraps.

Figure S7. *Stability of the edges of individual IGD symptoms – Girls, Czechia*

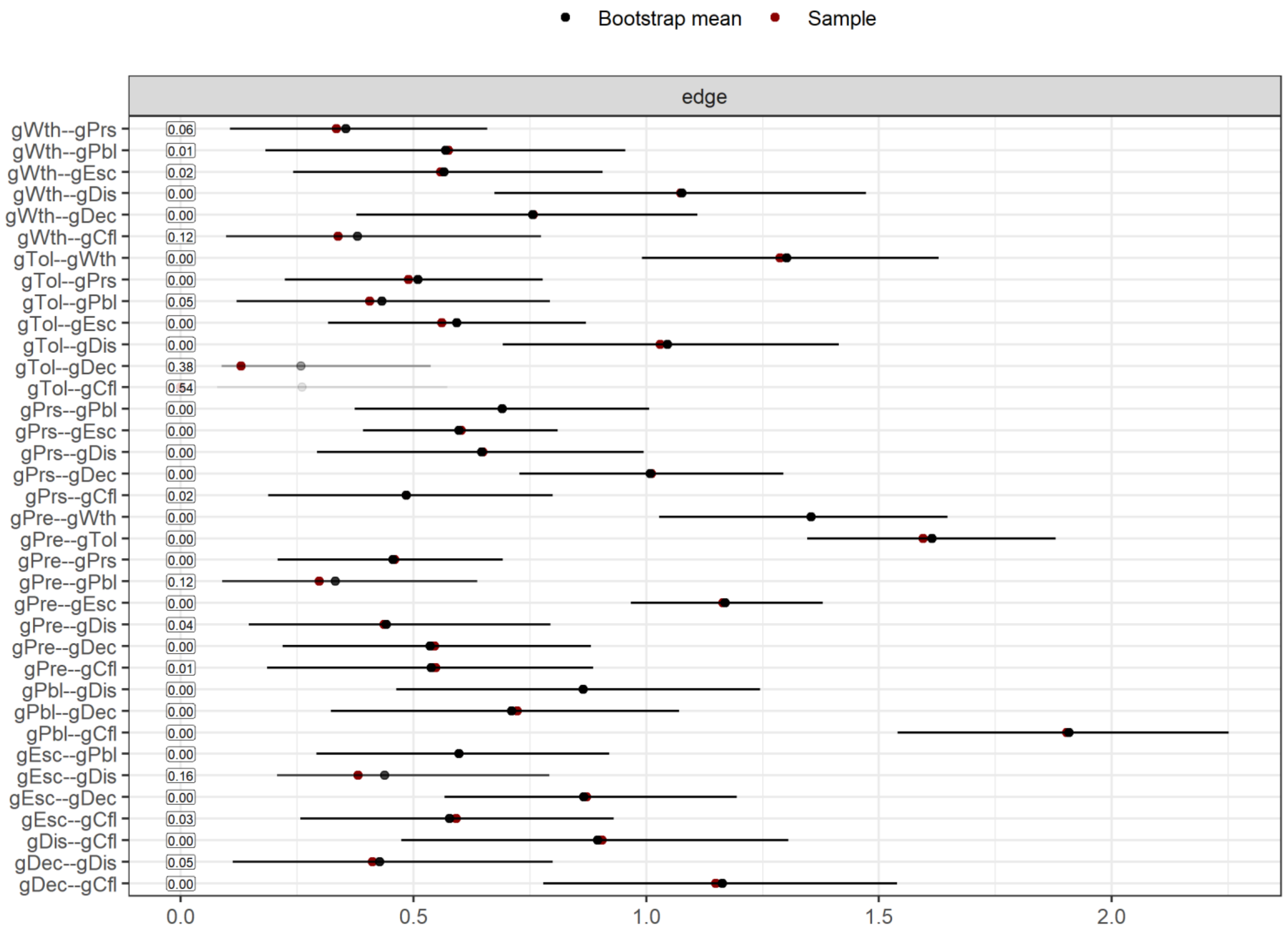

Note: The numbers in rectangles show how often an edge was estimated non-zero in the 1000 bootstraps.

Figure S8. *Stability of the edges of individual IGD symptoms – Boys, England*

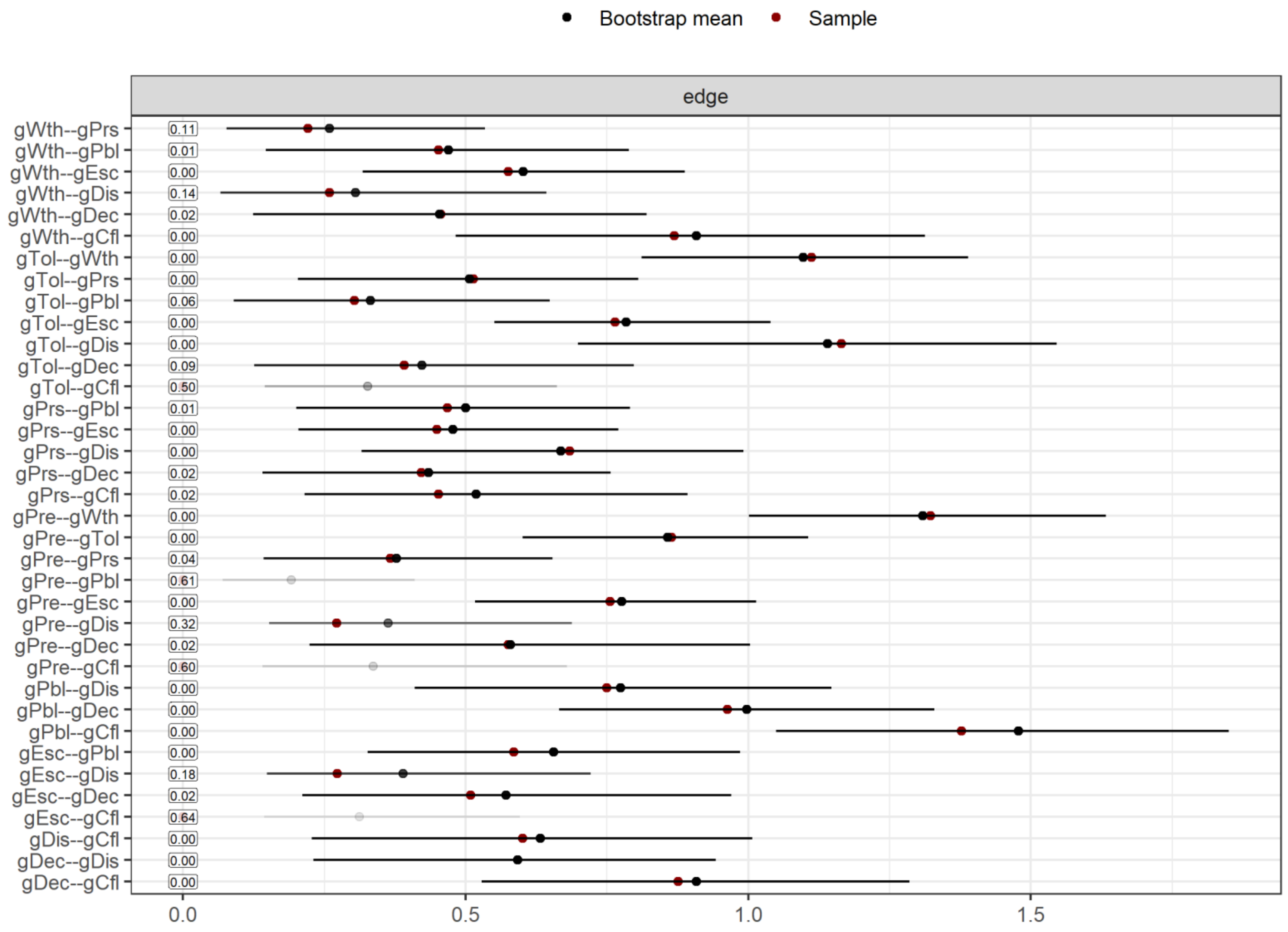

Note: The numbers in rectangles show how often an edge was estimated non-zero in the 1000 bootstraps.

Figure S9. *Stability of the edges of individual IGD symptoms – Girls, England*

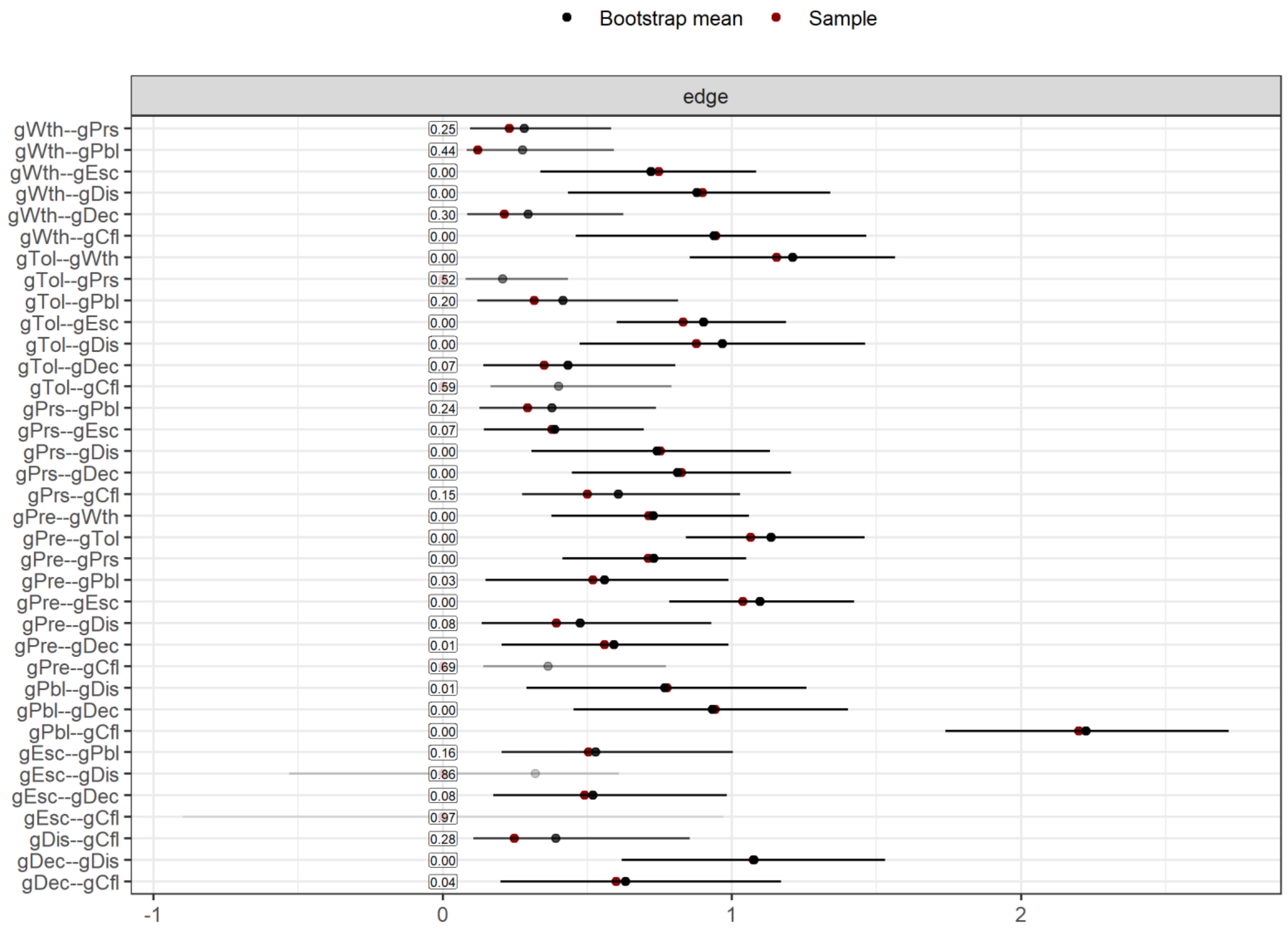

Note: The numbers in rectangles show how often an edge was estimated non-zero in the 1000 bootstraps.

Figure S10. *Stability of the edges of individual IGD symptoms – Boys, Estonia*

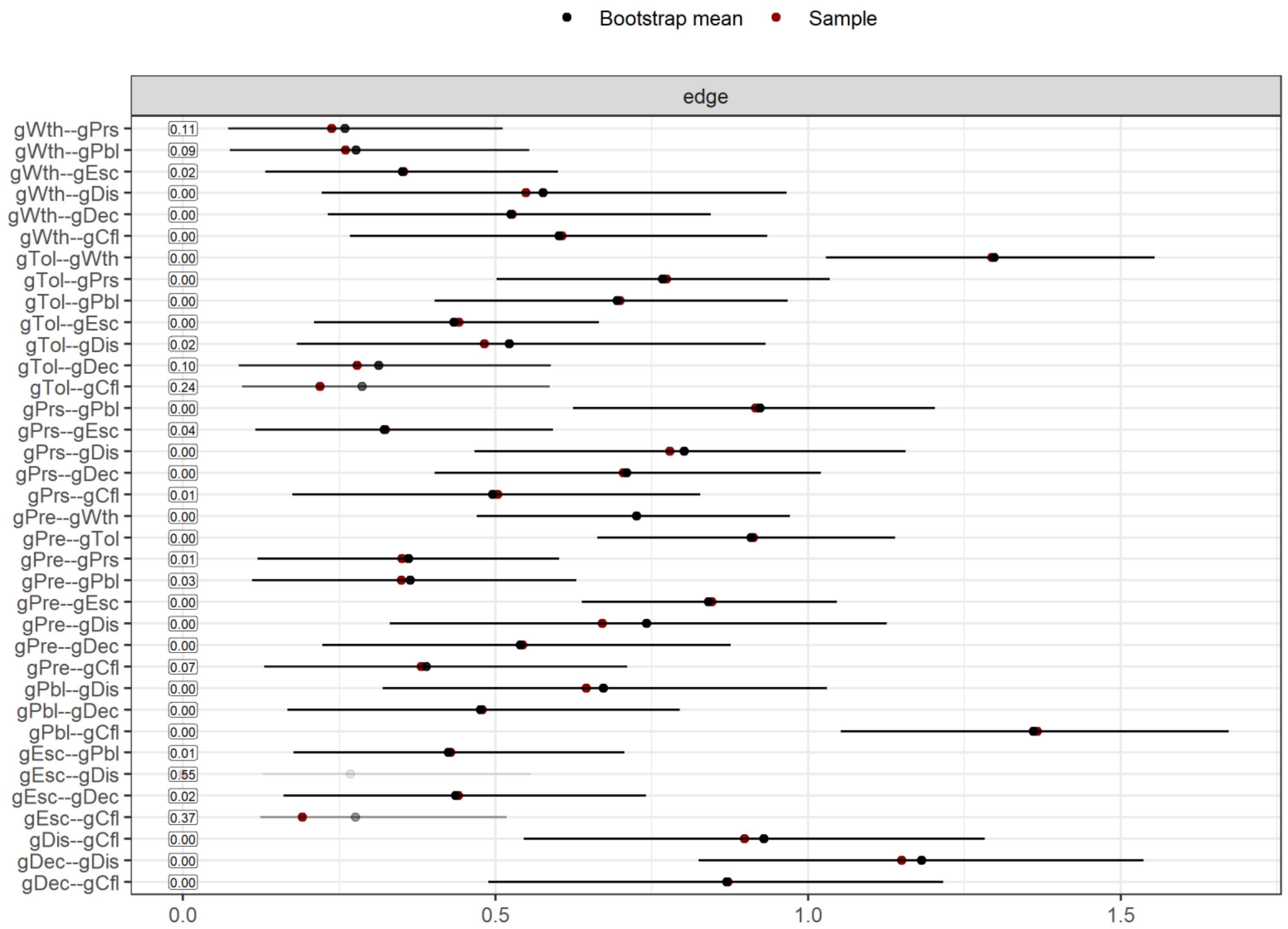

Note: The numbers in rectangles show how often an edge was estimated non-zero in the 1000 bootstraps.

Figure S11. *Stability of the edges of individual IGD symptoms – Girls, Estonia*

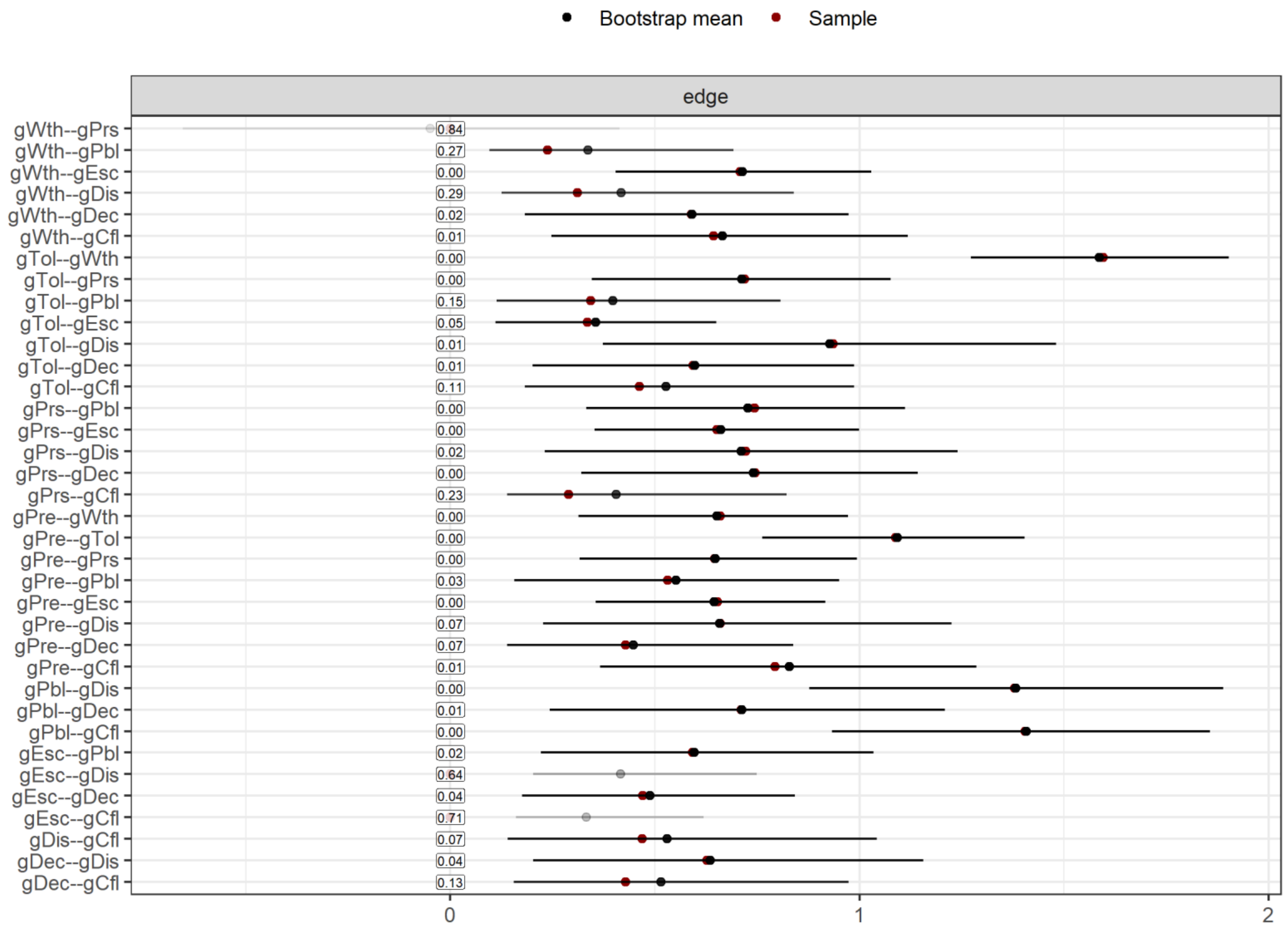

Note: The numbers in rectangles show how often an edge was estimated non-zero in the 1000 bootstraps.

Figure S12. *Stability of the edges of individual IGD symptoms – Boys, Iceland*

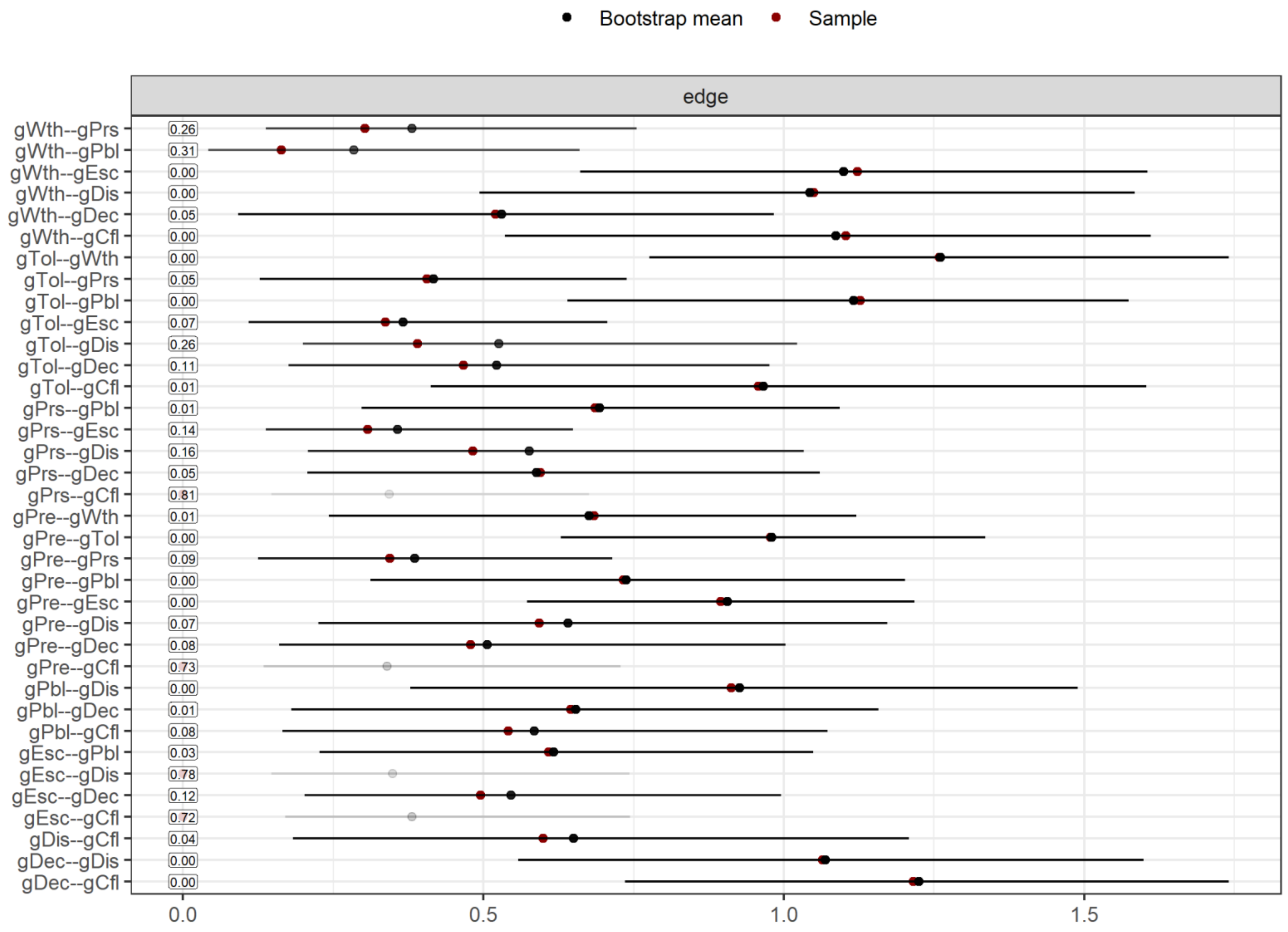

Note: The numbers in rectangles show how often an edge was estimated non-zero in the 1000 bootstraps.

Figure S13. *Stability of the edges of individual IGD symptoms – Girls, Iceland*

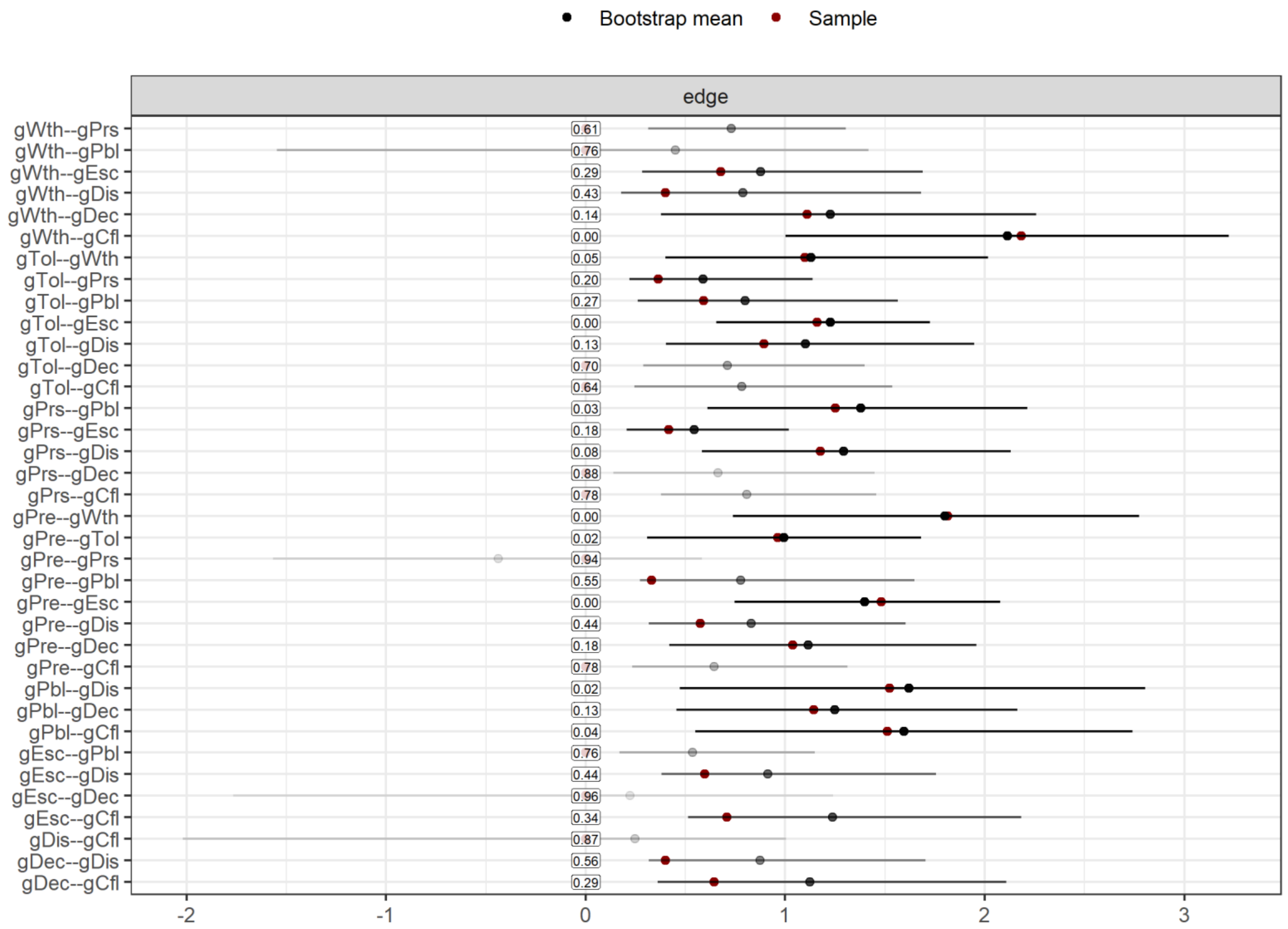

Note: The numbers in rectangles show how often an edge was estimated non-zero in the 1000 bootstraps.

Figure S14. *Stability of the edges of individual IGD symptoms – Boys, Malta*

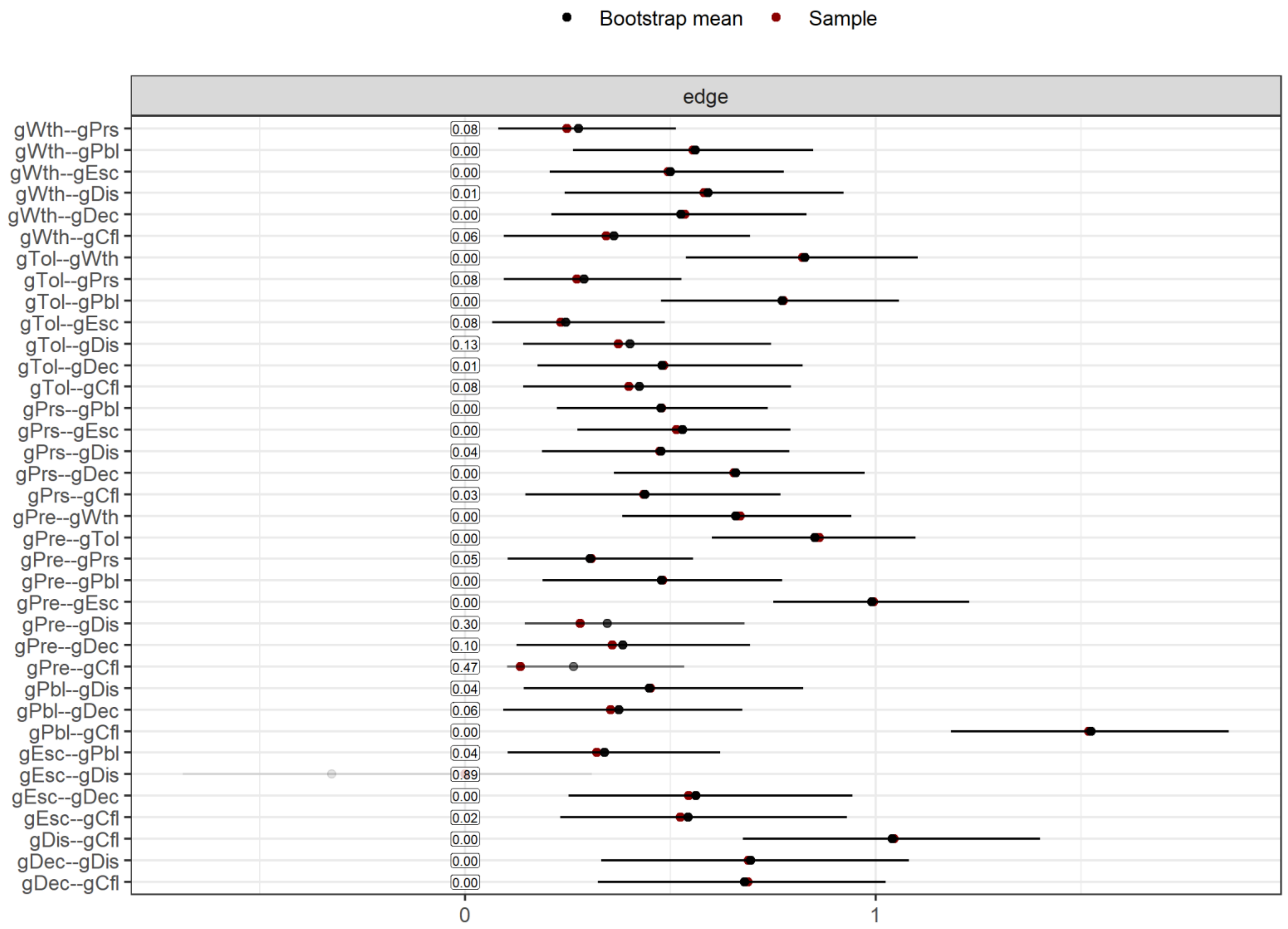

Note: The numbers in rectangles show how often an edge was estimated non-zero in the 1000 bootstraps.

Figure S15. *Stability of the edges of individual IGD symptoms – Girls, Malta*

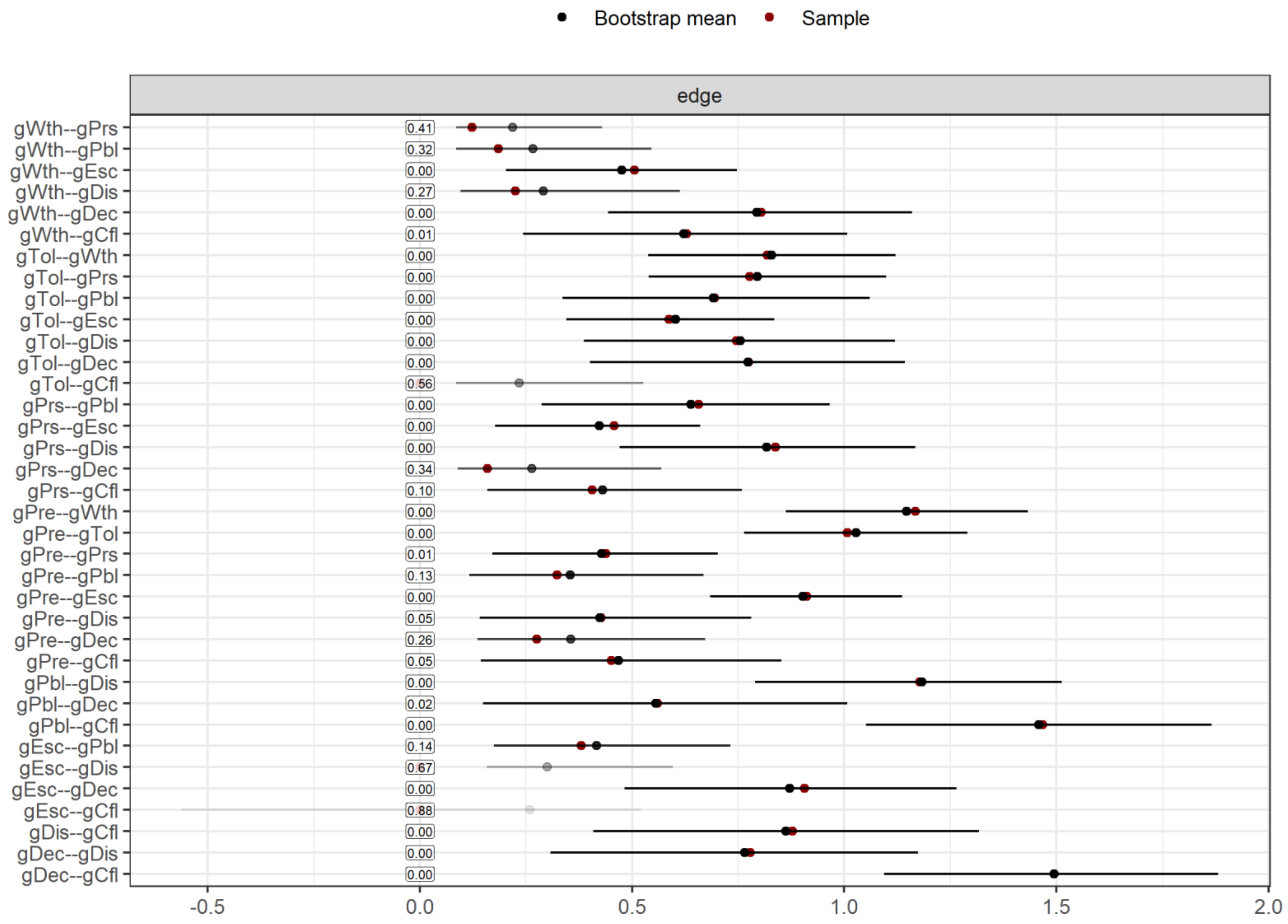

Note: The numbers in rectangles show how often an edge was estimated non-zero in the 1000 bootstraps.

Figure S16. *Stability of the edges of individual IGD symptoms – Boys, Netherlands*

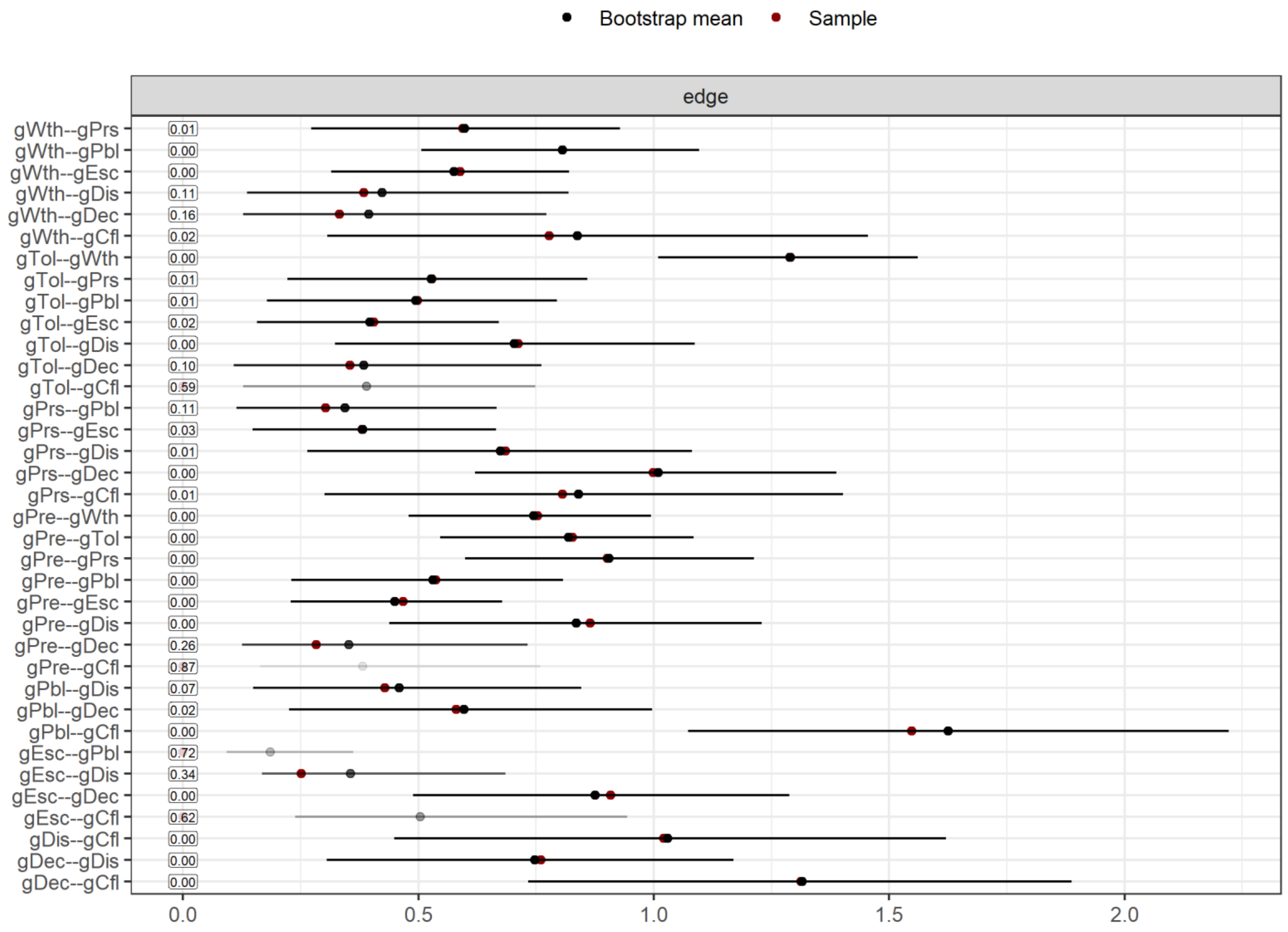

Note: The numbers in rectangles show how often an edge was estimated non-zero in the 1000 bootstraps.

Figure S17. *Stability of the edges of individual IGD symptoms – Girls, Netherlands*

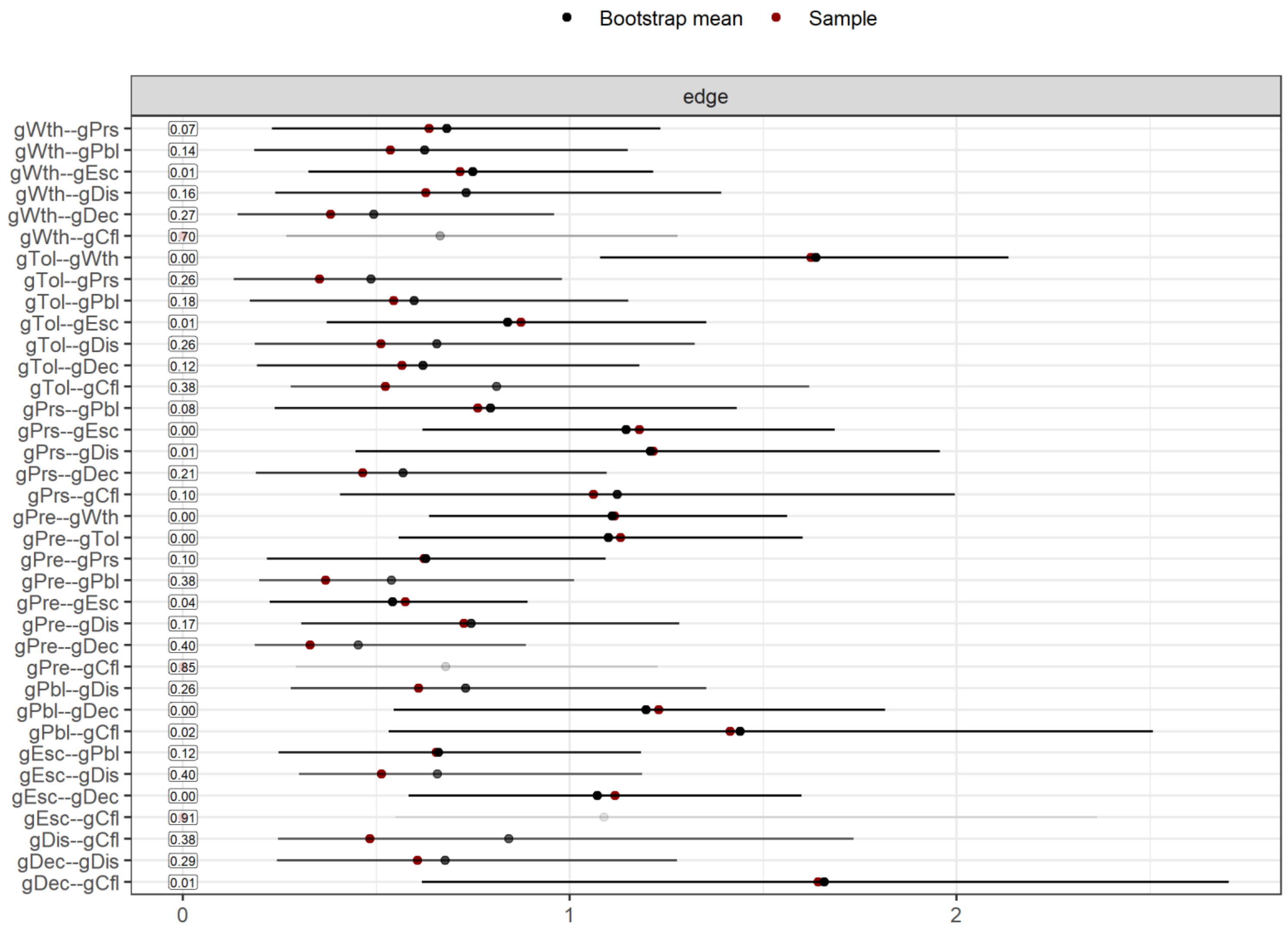

Note: The numbers in rectangles show how often an edge was estimated non-zero in the 1000 bootstraps.

Figure S18. *Stability of the edges of individual IGD symptoms – Boys, North Macedonia*

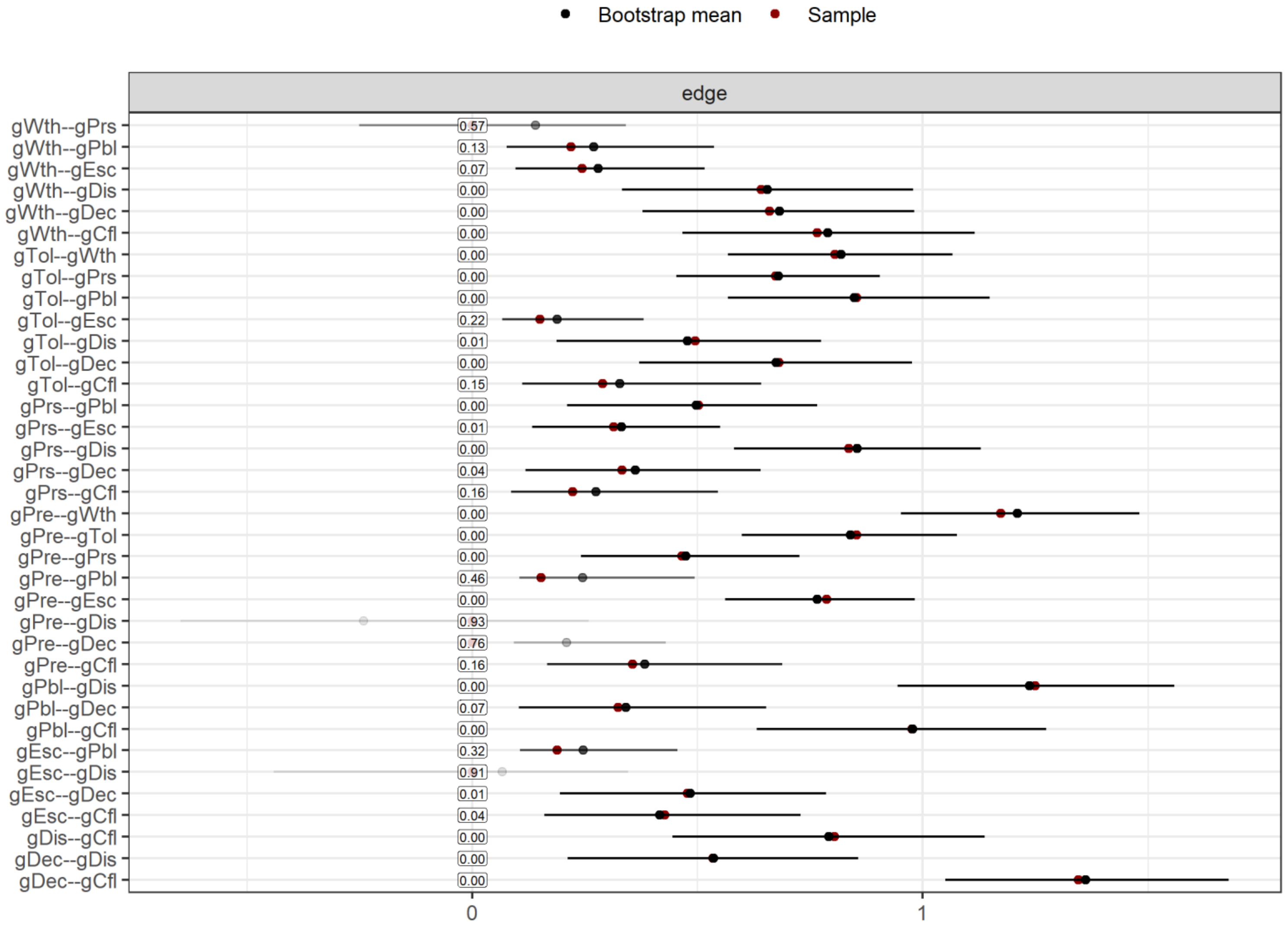

Note: The numbers in rectangles show how often an edge was estimated non-zero in the 1000 bootstraps.

Figure S19. *Stability of the edges of individual IGD symptoms – Girls, North Macedonia*

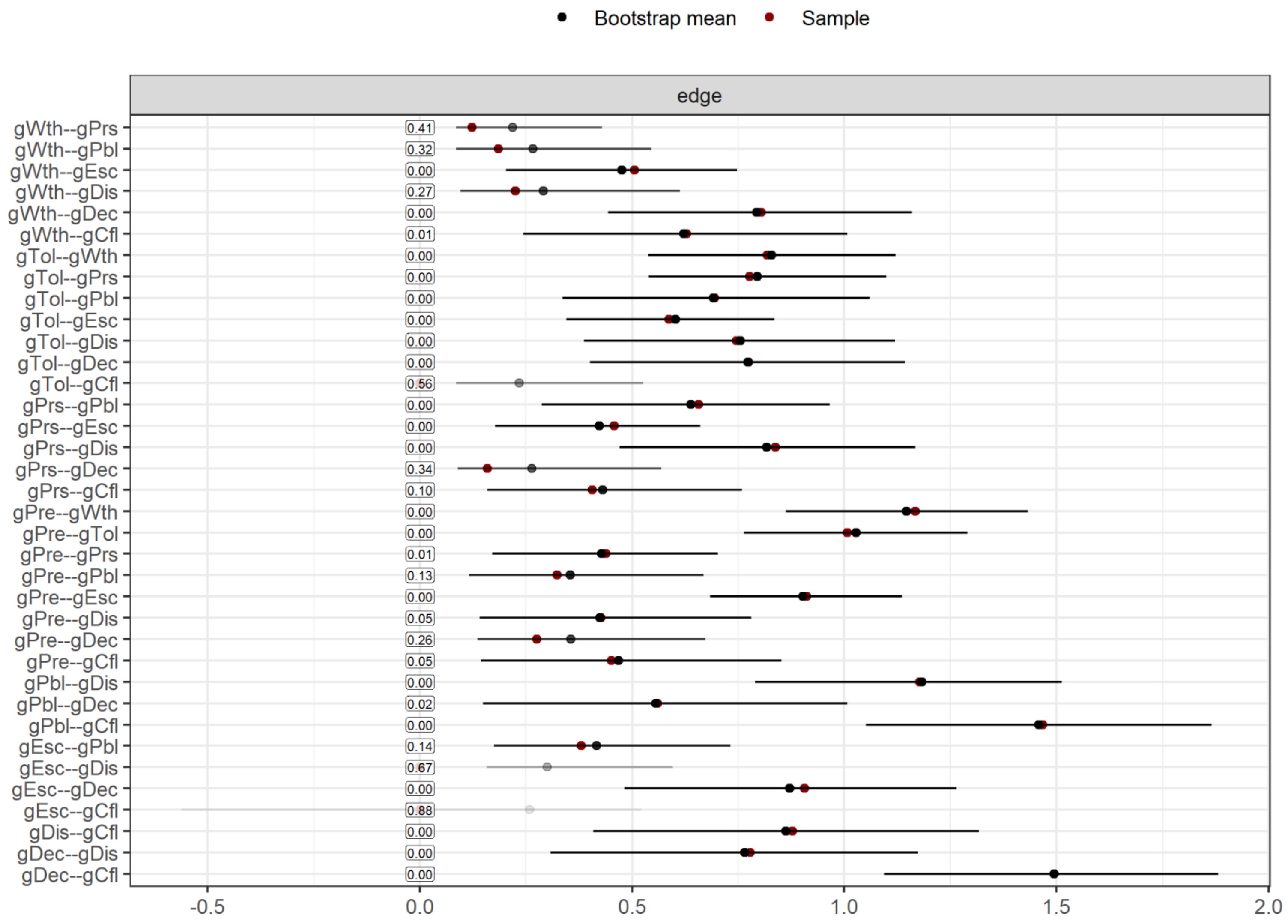

Note: The numbers in rectangles show how often an edge was estimated non-zero in the 1000 bootstraps.

Figure S20. *Stability of the edges of individual IGD symptoms – Boys, Scotland*

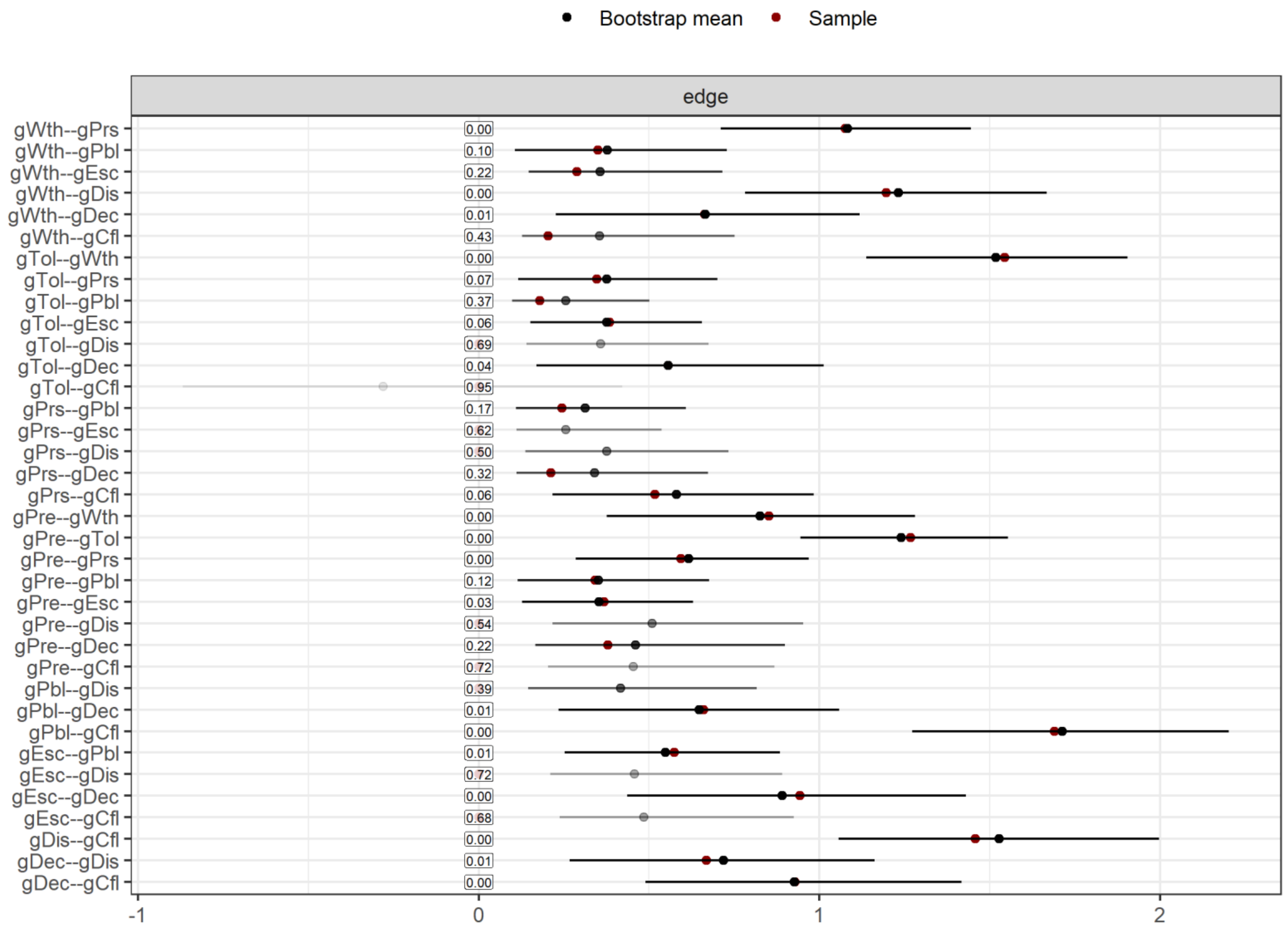

Note: The numbers in rectangles show how often an edge was estimated non-zero in the 1000 bootstraps.

Figure S21. *Stability of the edges of individual IGD symptoms – Girls, Scotland*

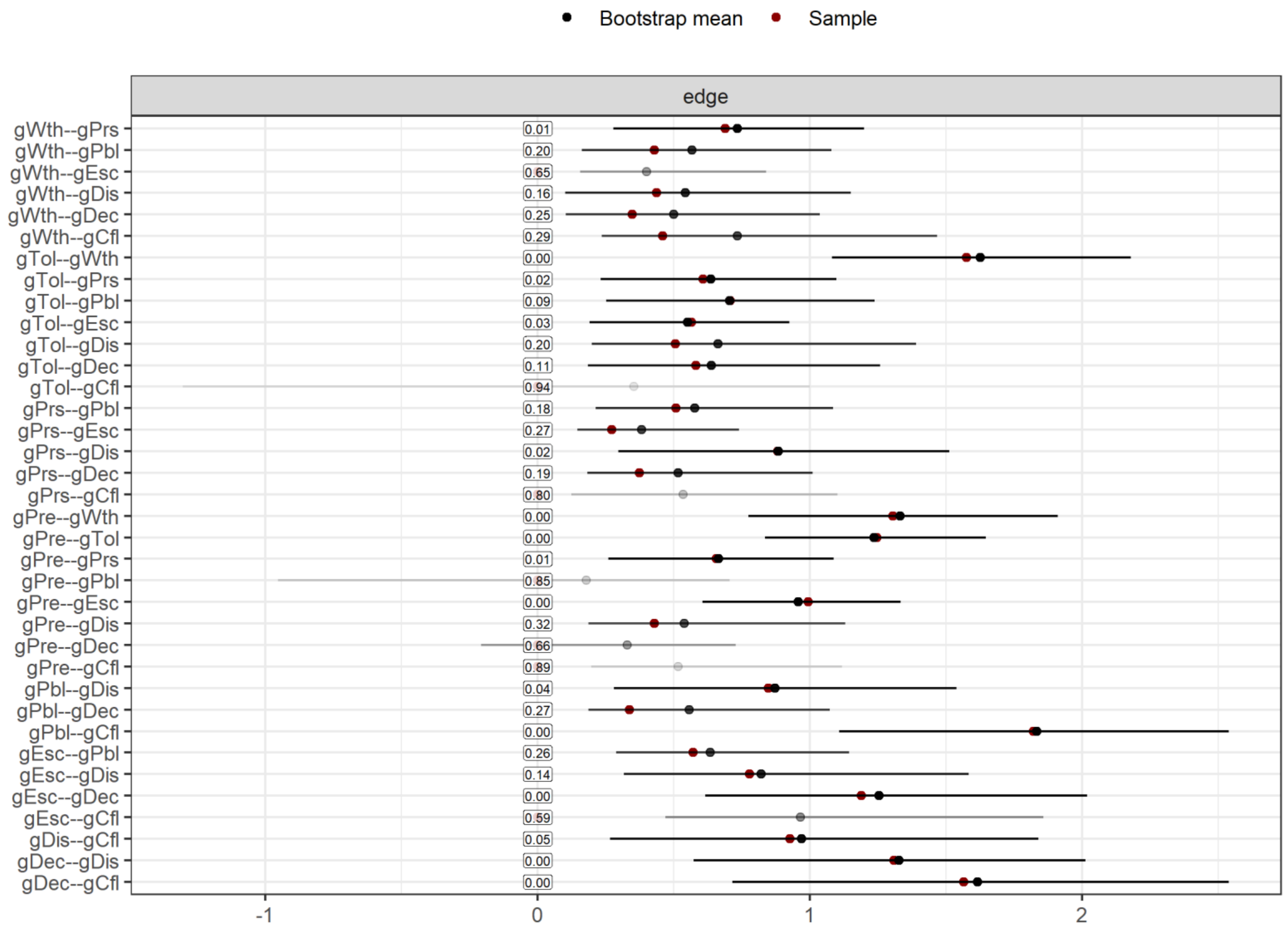

Note: The numbers in rectangles show how often an edge was estimated non-zero in the 1000 bootstraps.

Figure S22. *Stability of the edges of individual IGD symptoms – Boys, Serbia*

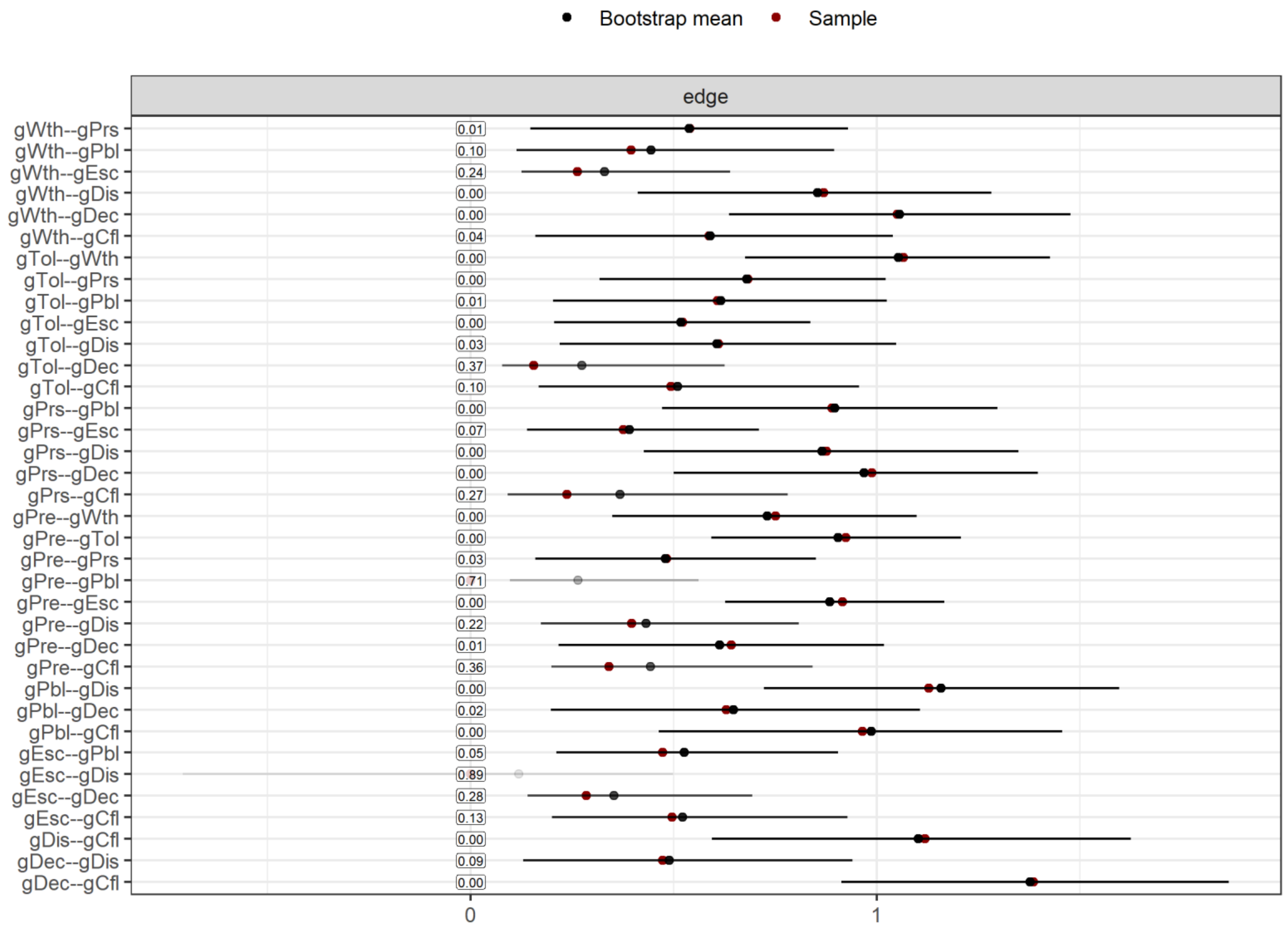

Note: The numbers in rectangles show how often an edge was estimated non-zero in the 1000 bootstraps.

Figure S23. *Stability of the edges of individual IGD symptoms – Girls, Serbia*

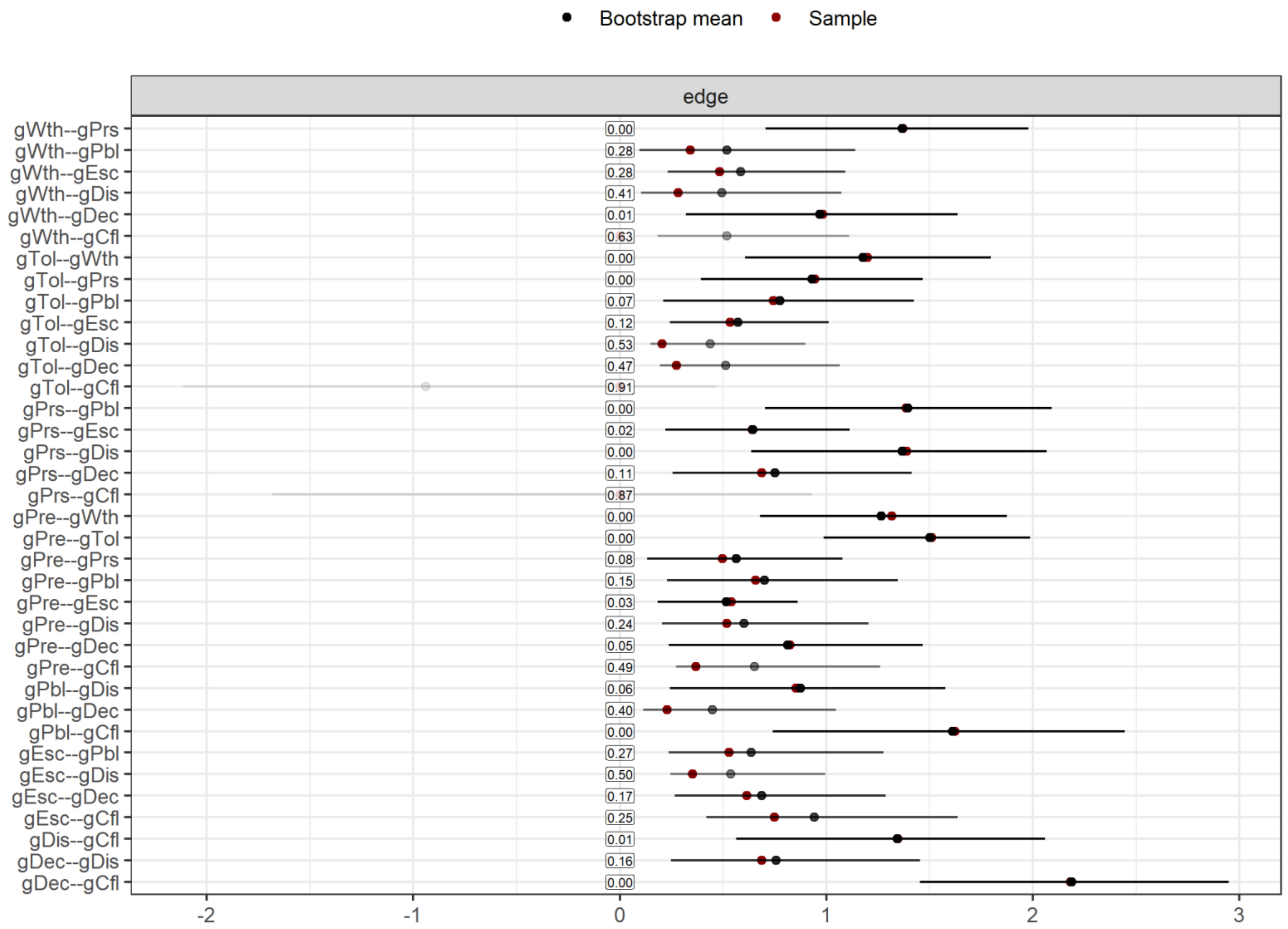

Note: The numbers in rectangles show how often an edge was estimated non-zero in the 1000 bootstraps.

Figure S24. *Stability of the edges of individual IGD symptoms – Boys, Slovakia*

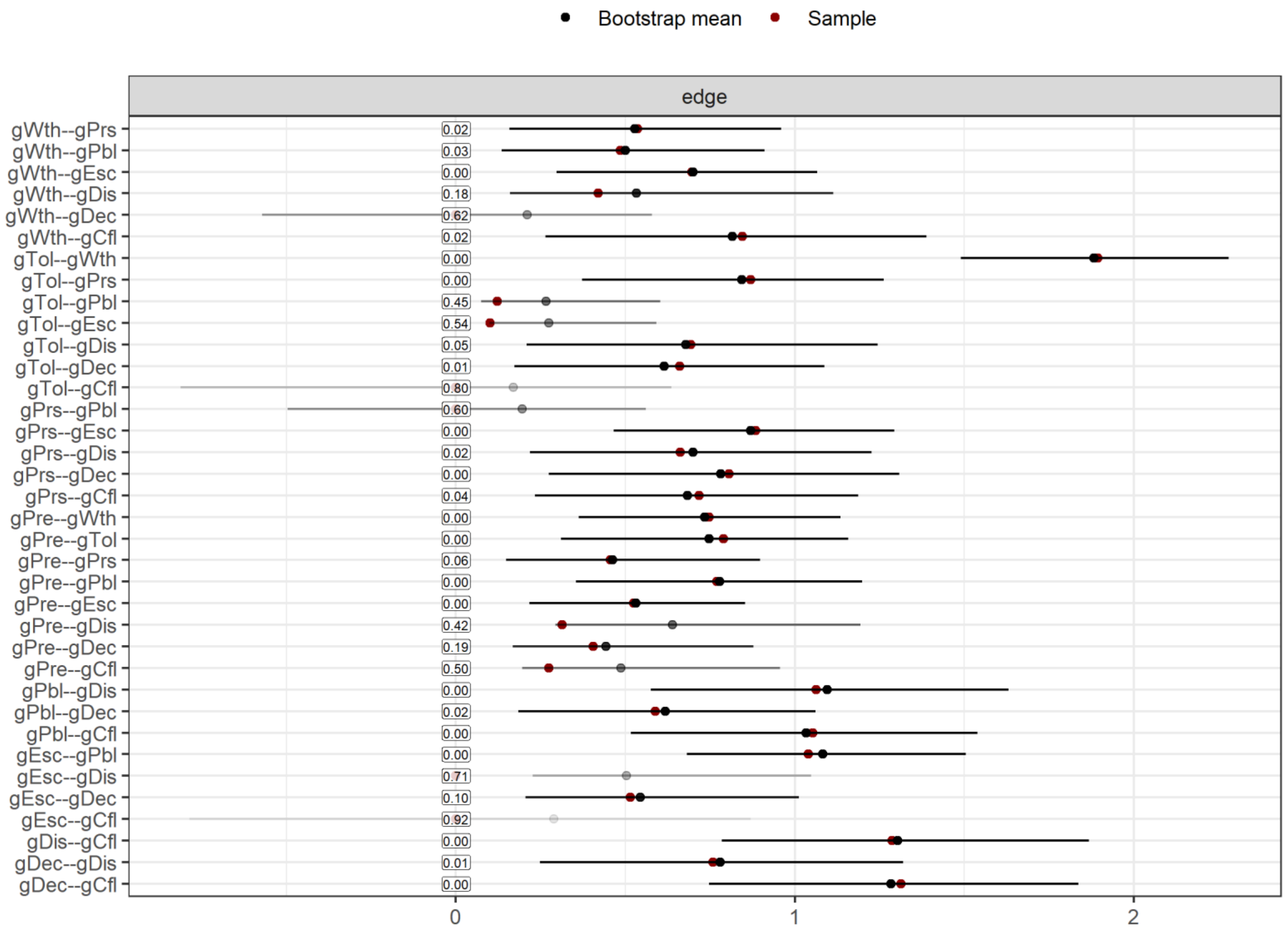

Note: The numbers in rectangles show how often an edge was estimated non-zero in the 1000 bootstraps.

Figure S25. *Stability of the edges of individual IGD symptoms – Girls, Slovakia*

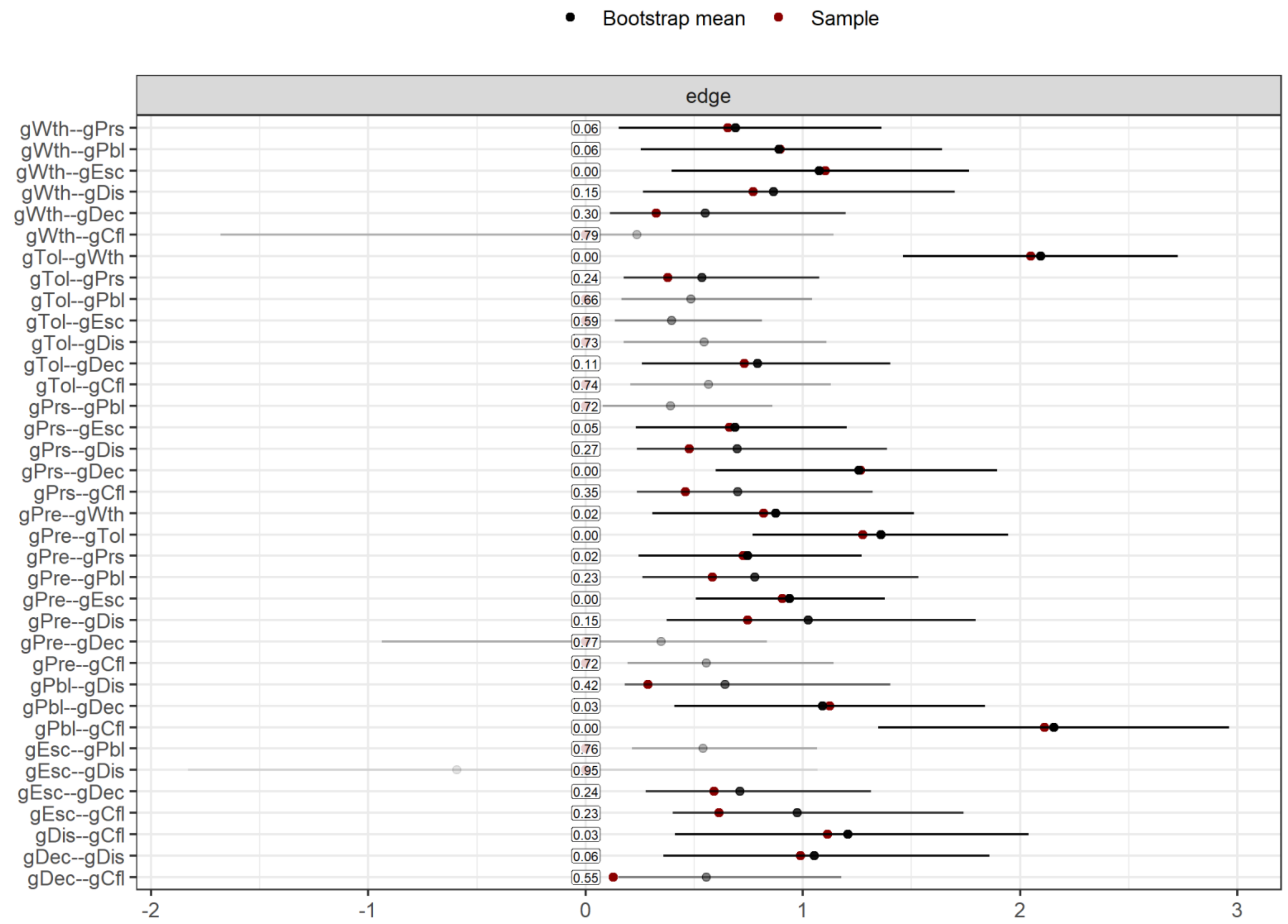

Note: The numbers in rectangles show how often an edge was estimated non-zero in the 1000 bootstraps.

Figure S26. *Stability of the edges of individual IGD symptoms – Boys, Slovenia*

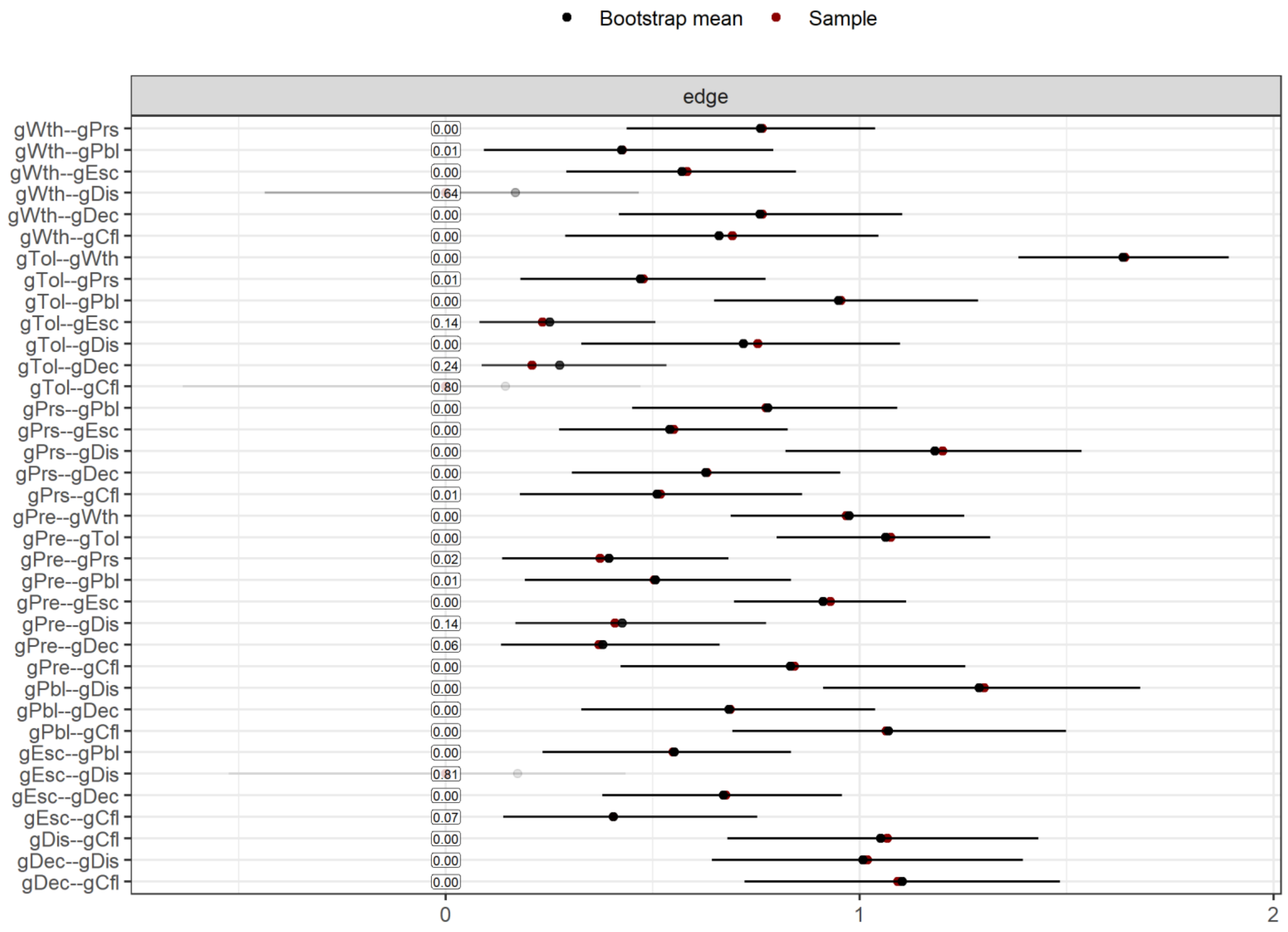

Note: The numbers in rectangles show how often an edge was estimated non-zero in the 1000 bootstraps.

Figure S27. *Stability of the edges of individual IGD symptoms – Girls, Slovenia*

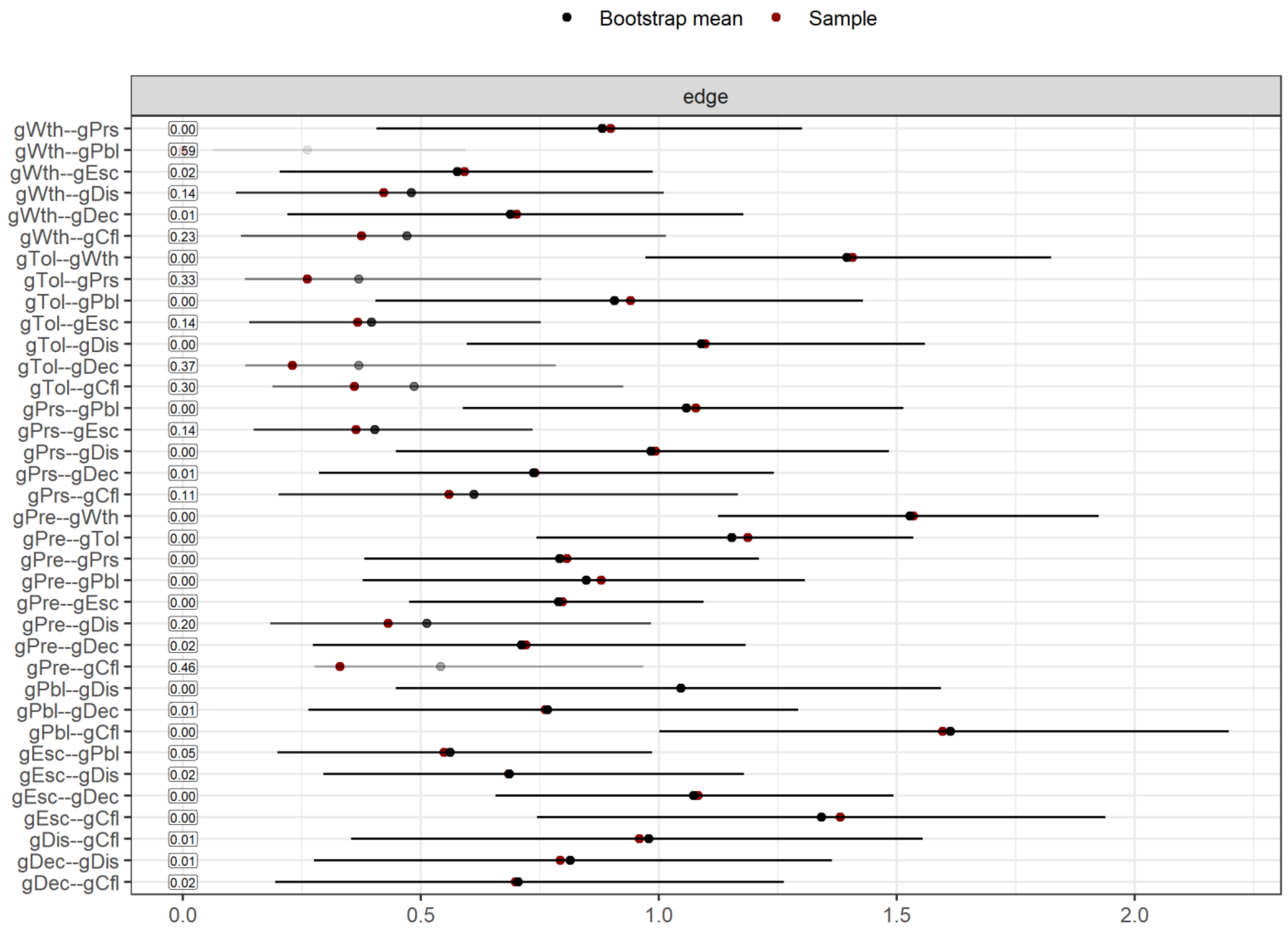

Note: The numbers in rectangles show how often an edge was estimated non-zero in the 1000 bootstraps.
